# A Phosphanyl‐Phosphagallene that Functions as a Frustrated Lewis Pair

**DOI:** 10.1002/anie.202008207

**Published:** 2020-09-09

**Authors:** Daniel W. N. Wilson, Joey Feld, Jose M. Goicoechea

**Affiliations:** ^1^ Department of Chemistry University of Oxford Chemistry Research Laboratory 12 Mansfield Road Oxford OX1 3TA UK

**Keywords:** decarbonylation, gallium, frustrated Lewis pairs, phosphaketenes, phosphorus

## Abstract

Phosphagallenes (**1 a**/**1 b**) featuring double bonds between phosphorus and gallium were synthesized by reaction of (phosphanyl)phosphaketenes with the gallium carbenoid Ga(Nacnac) (Nacnac=HC[C(Me)N(2,6‐*i*‐Pr_2_C_6_H_3_)]_2_). The stability of these species is dependent on the saturation of the phosphanyl moiety. **1 a**, which bears an unsaturated phosphanyl ring, rearranges in solution to yield a spirocyclic compound (**2**) which contains a P=P bond. The saturated variant **1 b** is stable even at elevated temperatures. **1 b** behaves as a frustrated Lewis pair capable of activation of H_2_ and forms a 1:1 adduct with CO_2_.

## Graphical abstract

We describe the synthesis of compounds exhibiting gallium–phosphorus double bonds. The stability of these species is dependent on the saturation of the phosphanyl moiety, and the saturated variant behaves as a frustrated Lewis pair that is capable of activating H_2_ and forms a 1:1 adduct with CO_2_.

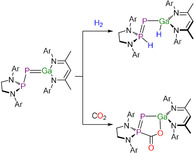

Once thought inaccessible, multiple bonds involving main group elements with a principal quantum number (*n*) greater than 2 have been of interest for decades.^[1]^ The inherent weakness of these bonds, which is partly due to ineffective p_π_‐p_π_ orbital overlap, gives rise to reactivity that contrasts with that of their lighter analogues. Heteroatomic multiple bonds between group 13/15 elements are of particular interest due to their valence isoelectronic relationship to C−C bonds. The behaviour of such species is exemplified by compounds containing B=N bonds, which display electronic properties and reactivity that differ significantly from C=C bonds. For example, incorporation of B=N units into aromatic systems has been used for the preparation of materials with unique photophysical and electrochemical properties.^[2]^ Additionally, compounds of the type (R)HN=BH(R′) (R/R′=H, alkyl, aryl) have been explored as potential hydrogen storage materials.^[3]^

Examples of compounds with E=E′ bonds in which one element has *n*>2 (i.e. E=Al, Ga and E′=N; or E=B and E′=P, As) are less common. Nöth and co‐workers reported the first boranylidenephosphane containing a B=P double bond by employing sterically demanding substituents on the boron atom and coordination of the phosphorus centre to a Lewis acid.[^4^, ^5^] This strategy was inverted by Power and co‐workers, who employed a sterically bulky terphenyl group at the pnictogen atom, in addition to the Lewis basic 4‐dimethylaminopyridine at the boron centre which gave access to compounds containing B=P and B=As bonds.^[6]^ Power also developed a synthetic strategy allowing access to E=N (E=Al, Ga) bonds by employing a group 13 carbenoid E(Nacnac) (Nacnac=HC[C(Me)N(2,6‐*i*‐Pr_2_C_6_H_3_)]_2_) and sterically encumbered organic azides which liberate N_2_ to give the desired compounds.[^7^, ^8^] A similar strategy was recently utilised allowing access to anionic aluminium‐imides.[^8d^, ^8e^]

Heteroatomic multiple bonds between heavy group 13/15 elements are rarer due to their inherent weakness, and are prone to oligomerization. Von Hänisch and Hampe reported the dimeric [{Li(THF)_3_}_2_Ga_2_{As(Si^*i*^Pr_3_)}_4_] (**A**, Figure 1) through the reaction of GaCl_3_ with two equivalents of Li_2_As(Si^*i*^Pr_3_).^[9]^ More recently, the Schulz group reported the synthesis of the monomeric gallaarsene (**B**) by addition of two equivalents of the gallium carbenoid Ga(Nacnac) to Cp*AsCl_2_ (Cp*=C_5_Me_5_), in which one equivalent of Ga(Nacnac) acts as a sacrificial reductant.^[10]^ The same group also reported the first example of a gallastibene, (Nacnac)Ga=SbGa(Cl)(Nacnac) (**C**), by reduction of the radical [(Nacnac)(Cl)Ga]_2_Sb^.^ with KC_8_.^[11]^

**Figure 1:**
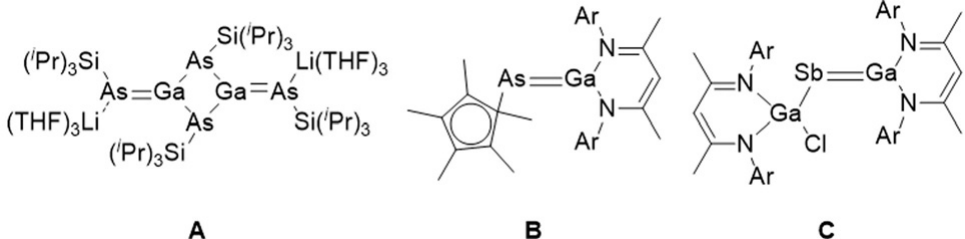
Previously reported examples of heteroatomic group 13/15 multiple bonds. Ar=2,6‐^*i*^Pr_2_C_6_H_3_.

Low‐valent species containing heavy group 13 and 15 elements are potential precursors to III/V semiconducting materials which have found applications in optoelectronic devices.^[12]^ With this in mind we aimed to expand the known synthetic pathways to access potential molecular precursors to such materials. The stability of **A**–**C** implies that the absence of structurally authenticated Ga=P (and indeed Al=P and In=P) bonds is likely due to the lack of a suitable synthetic pathway rather than the inherent instability of such compounds. Of particular interest is Power's ligand displacement strategy involving Ga(Nacnac) and azides. Phosphaketenes (RP=C=O) are isoelectronic to azides and are known to undergo decarbonylation processes, with a variety of Lewis bases.^[13]^ We reasoned that addition of the nucleophile Ga(Nacnac), to a phosphaketene would result in carbonyl displacement to yield a compound containing a Ga=P bond. For this study we selected [(HC)_2_(NAr)_2_P]PCO ([P]PCO) and [(H_2_C)_2_(NAr)_2_P]PCO ([SP]PCO) (Ar=2,6‐^*i*^Pr_2_C_6_H_3_) due to their previously reported, well‐behaved ligand‐substitution reactivity.^[14]^

Addition of Ga(Nacnac) to a solution of [P]PCO results in immediate effervescence, accompanied by a colour change from yellow to red (Scheme 1). The formation of [P]P=Ga(Nacnac) (**1 a**) is quantitative by NMR spectroscopy, as evidenced by the appearance of a new AX spin system in the ^31^P{^1^H} NMR spectrum displaying two doublet resonances at 176.6 and −43.0 ppm (^1^ *J*_P–P_=385 Hz) corresponding to the phosphanyl and phosphinidene centers, respectively. The former is comparable to that of the phosphaketene precursor, however the phosphinidene resonance is shifted to a higher frequency (cf. [P]PCO: ^31^P{^1^H} NMR=165.1 and −232.6 ppm; ^1^ *J*_P–P_=253 Hz), consistent with a decrease in shielding due to phosphorus lone pair donation into the gallium *p*‐orbital. Attempts to crystallize **1 a** by cooling a concentrated hexane solution to −35 °C resulted in a mixture of red and light‐yellow crystals. Monitoring a solution containing **1 a** by ^1^H and ^31^P{^1^H} NMR spectroscopy over 24 hours allows for observation of a new product, **2** (ca. 10 % conversion). Heating a solution containing **1 a** to 40 °C for 5 days allowed for complete conversion to **2**.

**Scheme 1:**
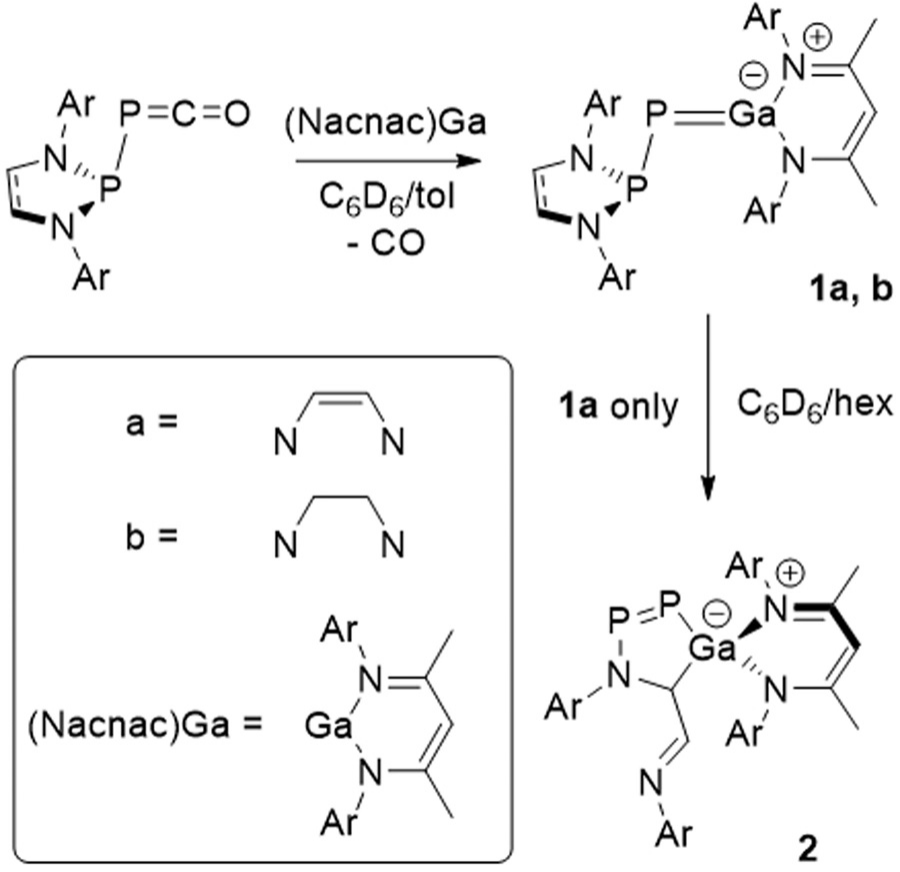
Synthesis of **1 a** and **1 b** through addition of Ga(Nacnac) (Nacnac=HC[C(Me)N(2,6‐*i*‐Pr_2_C_6_H_3_)]_2_ to (phosphanyl)phosphaketenes, and the rearrangement of **1 a** to **2**. Ar=2,6‐^*i*^Pr_2_C_6_H_3_.

Single crystal X‐ray diffraction studies performed on the red crystals confirms the identity of **1 a** (Figure 2). The crystal structure reveals a P1–Ga1 bond length of 2.165(1) Å, the *shortest* bond of its type reported to date. It is notably shorter than the sum of the double bond covalent radii for these elements [∑_*cov*_(P=Ga)=2.19 Å],^[15]^ consistent with significant P–Ga π‐bond character and/or a high degree of bond polarization as described by Su.^[16]^ The P1–P2 distance of 2.202(1) Å is significantly contracted with respect to that of [P]PCO (2.441(1) Å),^[14a]^ resulting in an increase of the ^1^ *J*_P–P_ coupling constant from 252 to 385 Hz.

**Figure 2:**
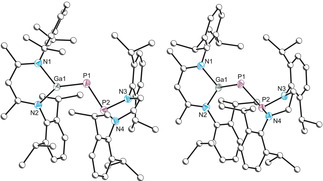
Molecular structure of **1 a** (left) and **1 b** (right). Ellipsoids set at 50 % probability; hydrogen atoms omitted for clarity. All carbon atoms are depicted as spheres of arbitrary radius. Selected interatomic distances [Å] and angles [°]: **1 a**: Ga1–P1 2.1650(7), P1–P2 2.2022(8), Ga1–N1 1.9186(19), Ga1–N2 1.941(2), P2–N3 1.7517(19), P2–N4 1.734(2); Ga1‐P1‐P2 101.31(3), N1‐Ga1‐N2 96.31(8), N4‐P2‐N3 86.58(9). **1 b**: Ga1–P1 2.1766(3), P1–P2 2.2119(4), Ga1–N1 1.9489(10), Ga1–N2 1.9280(11), P2–N3 1.7282(10), P2–N4 1.7343(10); Ga1‐P1‐P2 101.080(15), N2‐Ga1‐N1 95.96(5), N3‐P2‐N4 87.79(5).

The light‐yellow crystals were unambiguously identified as compound **2** (Figure 3), a constitutional isomer of **1 a**. It is likely formed from cleavage of one phosphanyl P−N bond and concomitant insertion of the Ga(Nacnac) group. The crystal structure displays a P–P bond length of 2.012(1) Å, in line with what is typically expected of a double bond (2.04 Å).^[15]^ The ^31^P{^1^H} NMR spectrum displays AX spin system with doublet resonances at 510.2 and 122.7 ppm which display a large ^1^ *J*_P–P_ coupling constant of 572 Hz. While we were unable to identify compounds analogous to **2** in the literature, a similar cyclic diphosphene was proposed as an intermediate in the rearrangement of [P]PCO.^[14a]^ A related benzo[*d*][1,2,3] azadiphosphole exhibits resonances at 246 and 354 ppm (^1^ *J*_P–P_=493 Hz).^[17]^ The discrepancy in the chemical shifts of these structurally similar compounds is likely due to a decrease of aromatic character in **2**, the heterocyclic core exhibits NICS(0) and NICS(1) values of 5.3 and 4.0, respectively, consistent with little aromatic character. The magnitude of the coupling constant of **2** is also greater, however comparable to linear diphosphenes such as (C_5_Me_5_)P=P(Ar′) (Ar′=2,4,6‐^*t*^Bu_3_C_6_H_2_) which displays a ^1^ *J*_P–P_ coupling of 584 Hz.^[18]^

**Figure 3:**
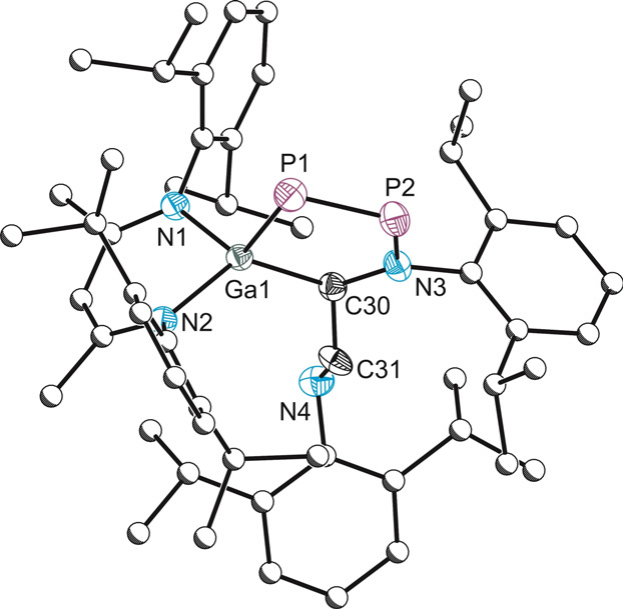
Molecular structure of **2**. Ellipsoids set at 50 % probability; hydrogen atoms omitted for clarity. All carbon atoms (with the exception of C30 and C31) are depicted as spheres of arbitrary radius. Selected interatomic distances [Å] and angles [°]: P1–P2 2.0121(10), P1–Ga1 2.3142(7), C1–Ga1 2.045(2), C2–N2 1.241(3), C1–N1 1.479(3), N1–P2 1.688(2), C1–C2 1.448(3), C1–N1 1.479(3); N1‐C1‐Ga1 108.28(15), P2‐P1‐Ga1 93.26(3), N1‐P2‐P1 111.94(8), N4‐Ga1‐N3 96.07(8).

Utilizing a phosphanyl with a saturated backbone, [SP]PCO, yields phosphaketenes with improved stability towards rearrangement.[^14b^, ^19^] Addition of Ga(Nacnac) to a solution of [SP]PCO in non‐coordinating solvents results in quantitative formation of [SP]P=Ga(Nacnac), **1 b** (Scheme 1). The ^31^P{^1^H} NMR spectrum displays two doublets at 157.8 and −61.3 ppm with a ^1^ *J*_P–P_ coupling of 346 Hz. As with **1 a**, the phosphinidene resonance is shifted to a significantly higher frequency (cf. [SP]PCO ^31^P{^1^H}=167.9 and −245.6 ppm; ^1^ *J*_P–P_=252 Hz). The ^1^H NMR spectrum is consistent with a single [SP] and Ga(Nacnac) moiety. It is notable that both **1 a** and **1 b** display three resonances corresponding to the isopropyl methine groups, indicative of free rotation about the P=Ga bond and a weak (3p‐4p)π‐bond. Monitoring a solution containing **1 b** by NMR spectroscopy indicated no rearrangement occurs, even upon heating to 80 °C. Crystals suitable for single crystal X‐ray diffraction were obtained from a concentrated hexane solution in moderate yields (50 %).

The crystal structure of **1 b** (Figure 2) reveals bond parameters comparable to **1 a**. The P1–Ga1 bond length is 2.177(1) Å, a small increase with respect to **1 a** but still below what is expected of a double bond. The P1–P2 bond length is also slightly elongated (2.212(1) Å).

Density functional theory (DFT) calculations were performed to better understand the electronic structure of **1 b**. Calculations were performed in the gas phase at the B3LYP level of theory using the basis sets Def2TZVP (Ga, P, N) and Def2SVP (C, H). The optimised structure, **1 b_DFT_**, displays bond parameters in good agreement to those of the solid‐state structure. The P1–P2 bond length is 2.257 Å (cf. 2.212(1) Å) and the P1–Ga1 bond length is 2.201 Å, a modest increase with respect to **1 b** (cf. 2.177(1) Å). The HOMO of **1 b_DFT_** primarily resides on both phosphorus lone pairs, while the HOMO−1 is mainly reflected by the π‐bonding interaction between Ga1 and P1 (Figure 4). Natural bond order analysis performed on the Ga=P bond reveals a σ‐bond (1.97e occupancy) composed of primarily *p*‐type (P; 14.55 % s, 84.54 % p) and s‐type (Ga; 83.97 % s, 15.89 % p) atomic orbitals. The Ga–P π‐bond (1.89e occupancy) is highly polarized towards the phosphorus center (82.74 % P) and is almost exclusively comprised of *p*‐orbital character (P 99.39 % p, Ga 99.53 % p). Natural population analysis further corroborates the polarized nature of this bond, with a highly electron deficient Ga1 (*q*=+1.30) and negative P1 (*q*=−0.80), while the phosphanyl P2 is positively charged (*q*=+0.96).

**Figure 4:**
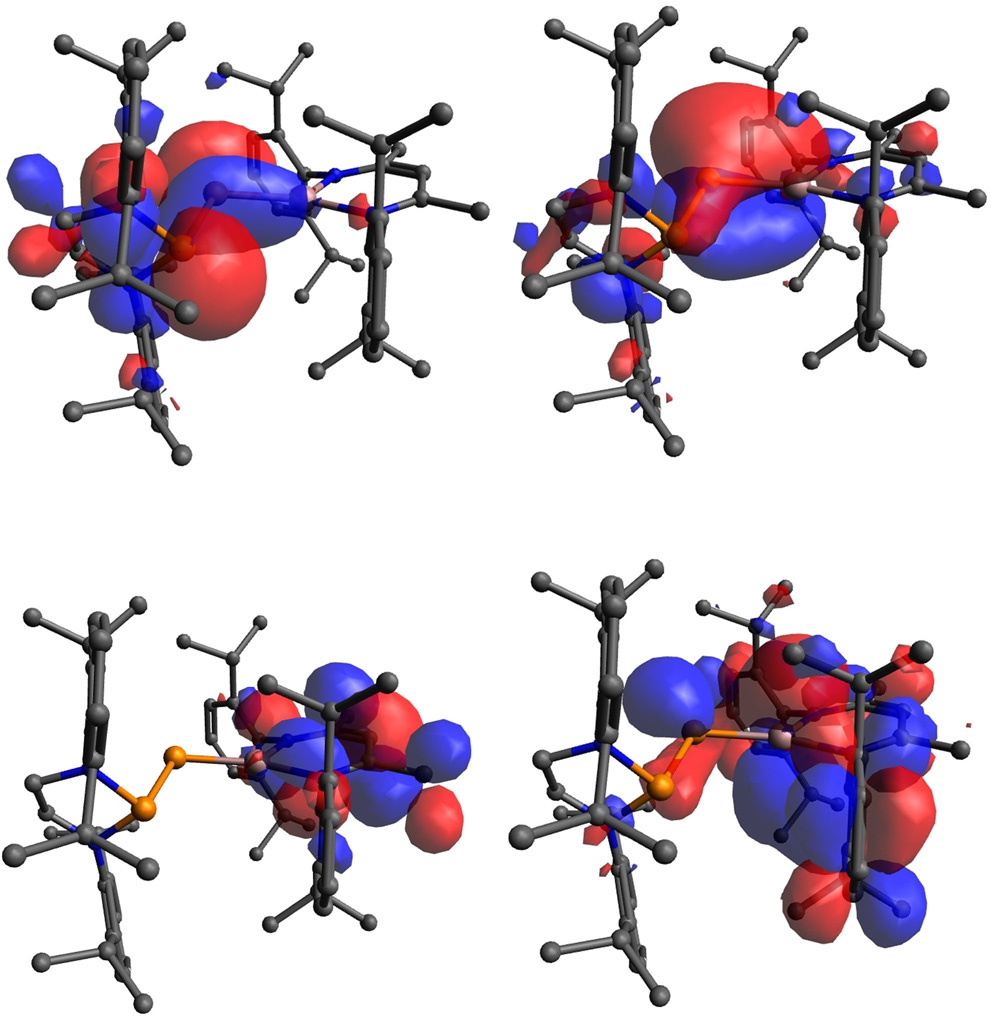
Top: HOMO (left) and HOMO−1. Bottom: LUMO (left) and LUMO+1 of **1 b_DFT_**.

We hypothesised that **1 b** may heterolytically cleave hydrogen due to the polarity of the Ga–P bond. Previous examples of homoatomic heavy element multiple bonds have shown that homolytic cleavage is possible, however to our knowledge there have been no examples of a heavy heteroatomic multiple bond capable of heterolytic hydrogen activation.[^20^, ^21^] Exposure of a solution containing **1 b** to 2 bar of H_2_ resulted in an immediate formation of **3** (Scheme 2). The ^31^P NMR spectrum indicated quantitative formation of a new product with a doublet of doublets resonance at 67.0 ppm (^1^ *J*_P–P_=578 Hz, ^1^ *J*_P–H_=457 Hz), corresponding to the phosphanyl phosphorus atom, and a broad doublet resonance at −248.7 ppm (^1^ *J*_P–P_=578 Hz), corresponding to the phosphinidene phosphorus atom. The former resonance collapses to a doublet upon proton decoupling. These data are consistent with protonation occurring exclusively at the phosphanyl phosphorus. The ^1^H NMR spectrum displays two new resonances, a doublet of doublets at 8.93 ppm with coupling to both phosphorus centers (^1^ *J*_H–P_=457 Hz, ^2^ *J*_H–P_=10 Hz) corresponding to the proton bound to the phosphanyl phosphorus and a broad singlet at 5.80 ppm. This latter resonance is in the expected region of a gallium hydride.^[22]^

**Scheme 2:**
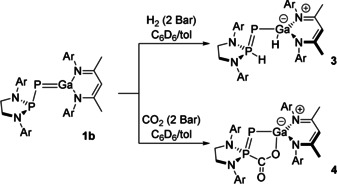
Synthesis of **3** and **4** by the reaction of **1 b** with H_2_ and CO_2_, respectively.

This unexpected reactivity can be rationalised as frustrated Lewis pair behaviour.^[23]^ The lone pair at the phosphanyl phosphorus (HOMO) acts in this case as the Lewis base. The π‐bond between the phosphinidene phosphorus and the gallium centre is sufficiently weak and polarized towards the phosphorus that the gallium *p*‐orbital is available to act as a Lewis acid (LUMO+1). The reaction is further aided by the generation of a P–P π‐bond at the expense of a significantly weaker Ga–P π‐bond.

Exposure of a solution containing **1 b** to an atmosphere of 2 bar carbon dioxide results in quantitative formation of **4** (Scheme 2), as evidenced by two new doublet resonances in the ^31^P{^1^H} NMR spectrum at 80.7 and −291.0 ppm (^1^ *J*_P–P_=588 Hz). The ^1^H NMR spectrum is consistent with a reduction in symmetry about the Ga(Nacnac) due to restriction in rotation upon formation of the heterocyclic core. The ^13^C NMR spectrum also displays a doublet of doublets resonance at 174.4 ppm with coupling to both phosphorus nuclei (^1^ *J*_C–P_=100 Hz, ^2^ *J*_C–P_=11 Hz). Crystals suitable for X‐ray diffraction were grown from a hexane solution at room temperature (74 % yield).

The crystal structure **4** confirms the formation of a CO_2_ adduct with **1 b**, with formation of new P–C and O–Ga bonds with bond lengths of 1.894(2) and 1.906(2) Å, respectively (Figure 5). The P1–P2 bond length of 2.064(1) Å is significantly contracted in comparison to **1 b**, falling in line with what is expected of a P–P double bond (∑_*cov*_(P=P)=2.04 Å).^[15]^ This is accompanied by elongation of the Ga1–P1 bond (2.297(1) Å) which falls in line with what is expected of a single bond [∑_*cov*_(P–Ga)=2.35 Å]. It is perhaps best to describe the activation process as a two‐electron oxidation of the phosphanyl phosphorus centre (**I**; Figure 6). A second, zwitterionic resonance form can be evoked with a formal positive charge on the phosphanyl site and negative charge on the phosphinidene (**II**), contrary to what is typical of FLP systems the negative charge is localized on the more electronegative P rather than the Ga (*χ*_Ga_=1.81, *χ*_P_=2.19). This resonance form is consistent with the low frequency ^31^P{^1^H} NMR resonance observed for this nucleus.

**Figure 5:**
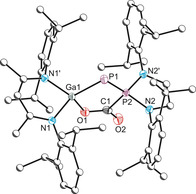
Molecular structure of **4**. Ellipsoids set at 50 % probability; hydrogen atoms omitted for clarity. All carbon atoms (with the exception of C1) are depicted as spheres of arbitrary radius. Selected interatomic distances [Å] and angles [°]: P1–Ga1 2.2971(7), P1–P2 2.0640(8), Ga1–O1 1.9061(18), Ga1–N1 1.9590(14), P2–N2 1.6861(15), P2–C1 1.894(2), O1–C1 1.282(3), O2–C1 1.227(3); P2‐P1‐Ga1 89.87(3), N2‐P2‐N2 91.36(10), N1‐Ga1‐N1 94.31(8), C1‐P2‐P1 109.57(9), O1‐Ga1‐P1 103.91(6), C1‐P2‐P1 109.57(9), O2‐C1‐O1 124.4(2), O2‐C1‐P2 116.2(2).

**Figure 6:**
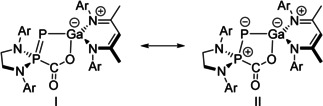
Resonances forms of **4**.

Adduct formation is not reversible under mild conditions, treatment of **4** under reduced pressure does not result in reformation of **1 b**. This contrasts with a geminal Ga/P FLP system reported by Uhl and co‐workers, which only showed a weak, reversible interaction with CO_2_ at low temperatures.^[24]^ It is likely the driving force of the forward reaction, the formation of strong σ‐bonds at the expense of a weak P–Ga π‐bond, provides a thermodynamic sink preventing the reverse process being accessible.

P/Ga FLPs capable of heterolytic cleavage of hydrogen have previously been limited to intermolecular systems in which the Ga feature electron withdrawing groups, that is, Ga(C_6_F_5_)_3_ with phosphines.^[25]^ To our knowledge, **1 b** represents the first P/Ga FLP to form an isolable adduct with CO_2_ and to activate H_2_ in an intramolecular fashion. It is also remarkable in that it does so with three π‐donating substituents adjacent to the Lewis‐acidic Ga centre.

In conclusion, we have synthesized a stable species containing a phosphorus‐gallium double bond, **1 b**, from a ligand exchange reaction between a phosphanyl‐phosphaketene and a gallium carbenoid. The reactivity of **1 b** towards H_2_ and CO_2_ was investigated, resulting in FLP type behaviour between the phosphanyl phosphorus and the gallium centre.

## Conflict of interest

The authors declare no conflict of interest.

## Supporting information

As a service to our authors and readers, this journal provides supporting information supplied by the authors. Such materials are peer reviewed and may be re‐organized for online delivery, but are not copy‐edited or typeset. Technical support issues arising from supporting information (other than missing files) should be addressed to the authors.

Supplementary
