# Supplementary material for: A Phosphanyl‐Phosphagallene that Functions as a Frustrated Lewis Pair
Source: Angew Chem Int Ed Engl. 2020 Sep 9;59(47):20914–8. doi: 10.1002/anie.202008207 (PMC7693089; doi:10.1002/anie.202008207)
Supplement: Supplementary file 1 — Supplementary [file ANIE-59-20914-s001.pdf]

Angewandte Chemie
Eine Zeitschrift der Gesellschaft Deutscher Chemiker

Supporting Information

A Phosphanyl-Phosphagallene that Functions as a Frustrated Lewis Pair

*Daniel W. N. Wilson, Joey Feld, and Jose M. Goicoechea**

anie_202008207_sm_miscellaneous_information.pdf

Supporting Information

A phosphanyl-phosphagallene that functions as a frustrated Lewis pair.

Daniel W. N. Wilson, Joey Feld and Jose M. Goicoechea*

Department of Chemistry, University of Oxford, Chemistry Research Laboratory, 12 Mansfield Road, Oxford, OX1 3TA, U.K.

E-mail: jose.goicoechea@chem.ox.ac.uk

Contents

1. Experimental section

2. Single crystal X-ray diffraction data

3. Computational details

4. References

1. Experimental Section

1.1. General synthetic methods

All reactions and product manipulations were carried out under an inert atmosphere of argon or dinitrogen using standard Schlenk-line or glovebox techniques (MBraun UNIlab glovebox maintained at < 0.1 ppm H_2O and < 0.1 ppm O_2). The phosphanylphosphaketenes, [P]PCO ([P] = $\{(CH)[(Dipp)N]\}_2P(PCO)$; Dipp = 2, 5-diisopropylphenyl), and [SP]PCO ([SP] = $\{(CH_2)[(Dipp)N]\}_2P(PCO)$; Dipp = 2, 5-diisopropylphenyl),[1,2] and the gallium carbenoid [(NacNac)Ga] (NacNac = $\{(Me)C[(Dipp)N]\}_2$),[3] were synthesized according to previously reported synthetic procedures. Hexane (hex; Sigma Aldrich, HPLC grade), and toluene (tol; Sigma Aldrich, HPLC grade) were purified using an MBraun SPS-800 solvent system. C_6D_6 (Aldrich, 99.5%) and d^8-toluene (Aldrich, 99.5%) were degassed prior to use. All dry solvents were stored under argon in gas-tight ampoules. All solvents were stored over 3 Å molecular sieves.

Analytical techniques: NMR spectra were acquired on a Bruker AVIII 500 MHz NMR spectrometer (^1H 500 MHz, ^{13}C 126 MHz) and a Bruker AVIII 400 MHz NMR spectrometer (^1H 400 MHz, ^{31}P 162 MHz). ^1H and ^{13}C NMR spectra were referenced to the most downfield solvent resonance (^1H NMR C_6D_6: δ = 7.16 ppm; ^{13}C NMR C_6D_6: δ = 188.06 ppm). ^{31}P, was externally referenced to an 85% solution of H_3PO_4 in H_2O. Elemental analyses were carried out by Elemental Microanalyses Ltd. (Devon, U.K.). Samples (approx. 5 mg) were submitted in sealed Pyrex ampoules.

1.2. Compound synthesis.

1.2.1. Synthesis of [P]PGa(NacNac) (**1a**) and detection of rearranged species (**2**).

Equimolar amounts of [(NacNac)Ga] (20 mg, 0.042 mmol) and phosphanylphosphaketene, [P]PCO, (21 mg, 0.042 mmol) were dissolved in deuterated benzene (0.5 mL). Immediate effervescence was observed, accompanied by a darkening of the solution from yellow to red. Complete consumption of both starting materials was confirmed by ^{1}H and ^{31}P NMR spectroscopy and conversion can be seen to be quantitative. Isolation of a pure sample of **1a** was not possible due to a subsequent rearrangement in solution over the course of a several days. However, it was possible to obtain partial NMR spectroscopic characterisation from the reaction mixture. Crystallisation by cooling a concentrated hexane solution to –35°C allowed for confirmation of the identity of **1a**, which crystallises alongside the rearranged product **2**.

^{1}H NMR (400 MHz, C_6D_6) δ (ppm) = 7.26–7.18 (m, 8H; Dipp C*H*), 7.04–7.12 (m, 4H; Dipp C*H*), 5.84 (s, 2H; {(NC*H*)$_2$}), 4.86 (s, 1H; NacNac γ-H), 4.38 (d sept, $^{3}J_{H–H}$ = 6.7 Hz, $^{5}J_{H–P}$ = 3.0 Hz, 2H; [P] Dipp {C*H*(CH$_3$)$_2$}), 3.49 (d sept, $^{3}J_{H–H}$ = 7.0 Hz, $^{5}J_{H–P}$ = 3.5 Hz, 2H; [P] Dipp {C*H*(CH$_3$)$_2$}), 2.82 (sept, $^{3}J_{H–H}$ = 7.0 Hz, 4H; NacNac Dipp {C*H*(CH$_3$)$_2$}), 1.40 (d, $^{3}J_{H–H}$ = 4.5 Hz, 12H; NacNac backbone C*H*$_3$ and [P] Dipp {CH(C*H*$_3$)$_2$}), 1.31 (d, $^{3}J_{H–H}$ = 6.7 Hz, 6H; [P] Dipp {CH(C*H*$_3$)$_2$}), 1.26 (d, $^{3}J_{H–H}$ = 6.7 Hz, 6H; [P] Dipp {CH(C*H*$_3$)$_2$}), 1.20 (d, $^{3}J_{H–H}$ = 6.8 Hz, 6H; [P] Dipp {CH(C*H*$_3$)$_2$}), 1.00 (two overlapping doublets, both with $^{3}J_{H–H}$ = 6.8 Hz, 24H; NacNac Dipp {CH(C*H*$_3$)$_2$}). **^{31}P{^{1}H} NMR** (162 MHz, C_6D_6) δ (ppm) = 174.6 (d, $^{1}J_{P–P}$ = 385.6 Hz; phosphanyl), –43.0 (d, $^{1}J_{P–P}$ = 385.6 Hz; phosphinidene).

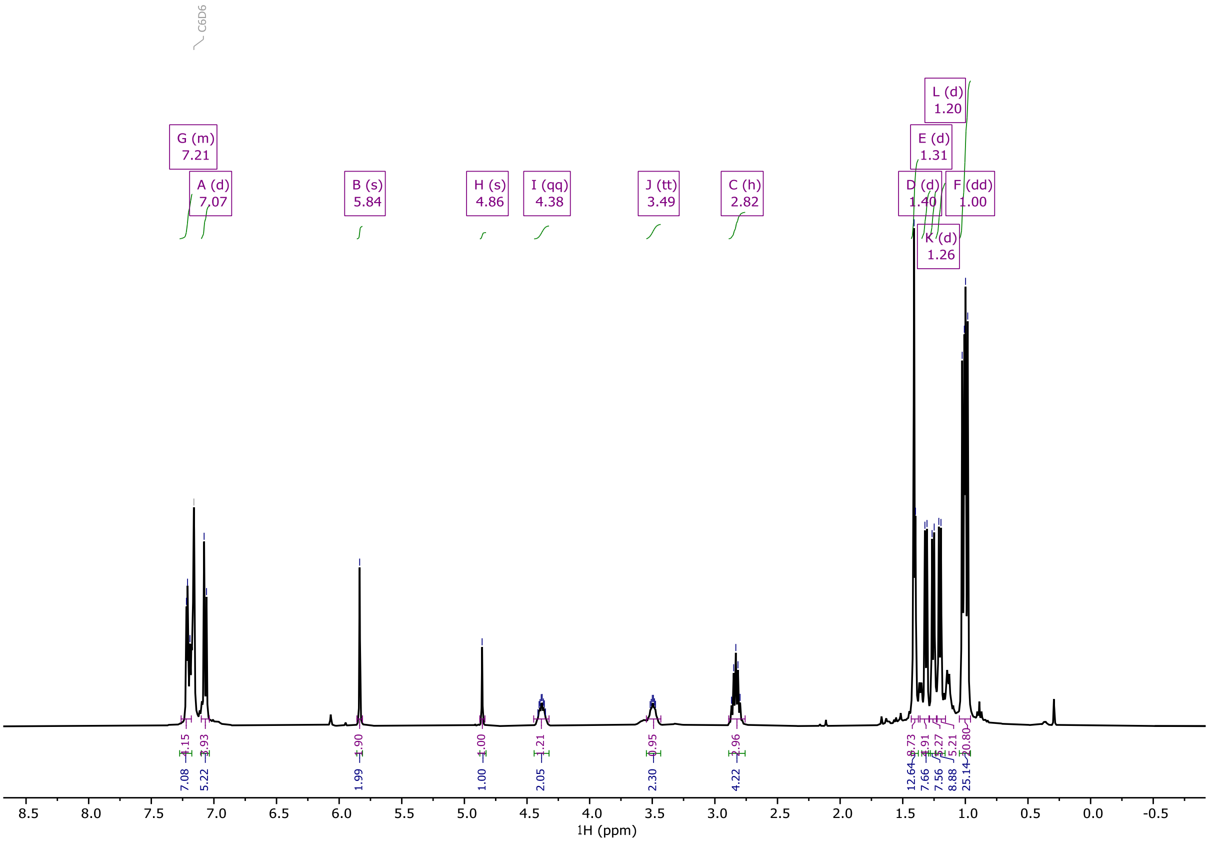

Figure S1. ^{1}H NMR spectrum of reaction mixture containing **1a** in C_6D_6.

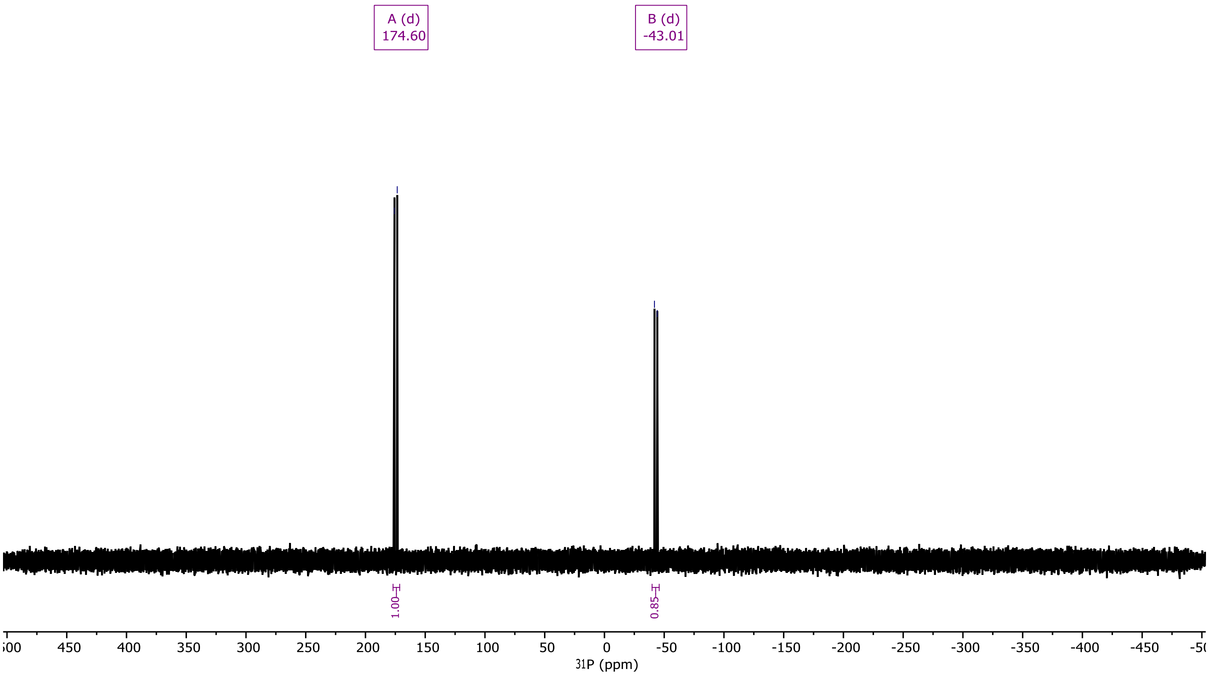

Figure S2. $^{31}P\{^{1}H\}$ NMR spectrum of reaction mixture containing **1a** in C_6D_6.

After 48 hours at –35°C rearranged compound **2** was observed by NMR spectroscopy:

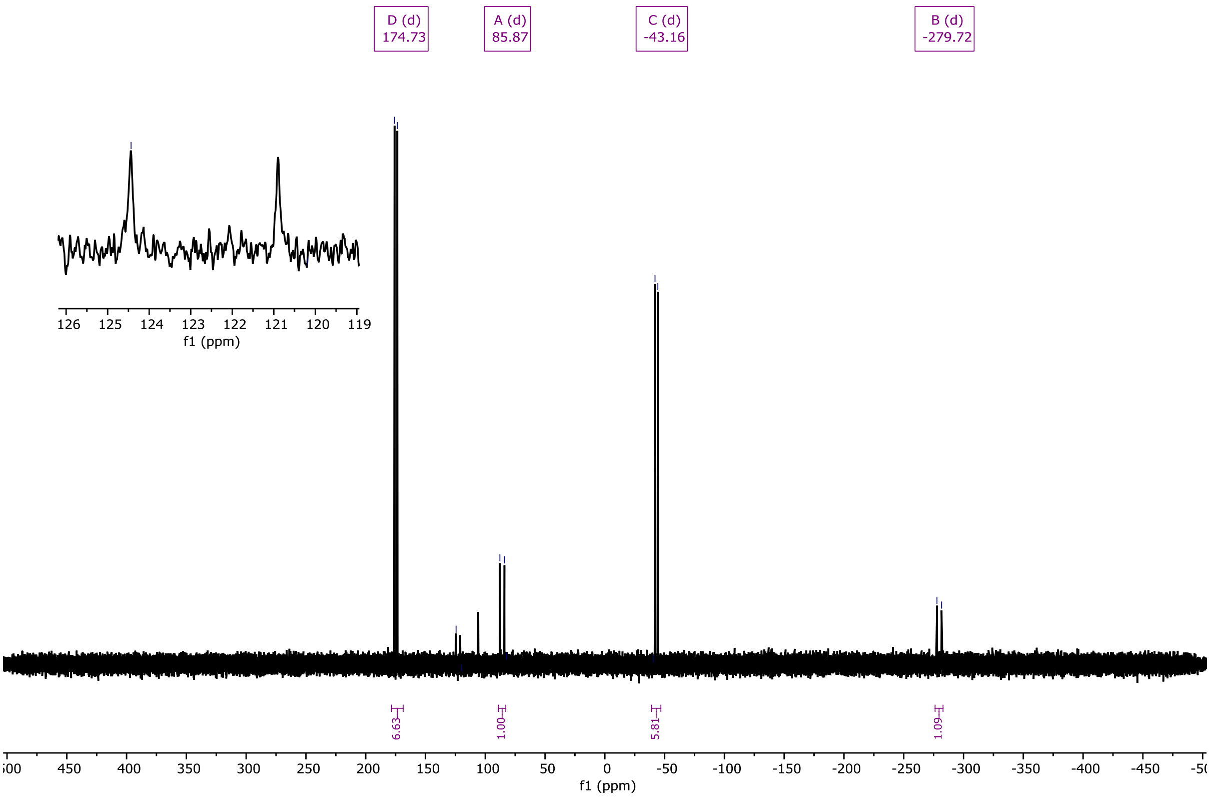

Figure S3. $^{31}P\{^{1}H\}$ NMR spectrum of reaction mixture containing **1a**, **2** (inset) and an unidentified species after 48 hours (C_6D_6).

1.2.2. Synthesis of **2**.

Equimolar amounts of [(NacNac)Ga] (20 mg, 0.042 mmol) and phosphanylphosphaketene, [P]PCO, (21 mg, 0.042 mmol) were dissolved in deuterated benzene (0.5 mL). Immediate effervescence was observed, accompanied by a darkening of the solution from yellow to red. The solution was heated to 40°C for 6 days, by which time complete consumption of **1a** could be observed by NMR spectroscopy. The solvent was removed under reduced pressure. Addition of hexane (1 mL) to the resulting orange oil resulted in formation of a pale-yellow precipitate. The suspension was cooled to –35°C for 3 hours and the hexane decanted. The yellow solid was dried under reduced pressure yielding **2** as a yellow powder. The decanted hexane was placed in the freezer to yield a second crop of **2** as pale-yellow crystals (combined yield 18mg, 46%)

^{1}H NMR (500 MHz, C_6D_6) δ = 7.86 (d, $^{3}J_{H–H}$ = 8.4 Hz, 1H, {NC*H*}), 7.14 – 6.92 (m, 12H, Ar*H*), 4.93 (s, 1H, NacNac γ-H), 4.15 (d, $^{3}J_{H–H}$ = 8.6 Hz, 1H, {NC([Ga])*H*}), 3.63 (sept, $^{3}J_{H–H}$ = 6.8 Hz, 1H, Dipp {C*H*(CH_3)$_2$}), 3.55 – 3.28 (m, 3H, Dipp {C*H*(CH_3)$_2$}),), 3.21 (sept, $^{3}J_{H–H}$ = 6.5 Hz, 1H, Dipp {C*H*(CH_3)$_2$}), 3.04 (sept, $^{3}J_{H–H}$ = 6.8 Hz, 1H, Dipp {C*H*(CH_3)$_2$}), 2.00 (sept, $^{3}J_{H–H}$ = 6.6 Hz, 2H, Dipp {C*H*(CH_3)$_2$}), 1.77 – 1.64 (m, 9H, NacNac C*H*$_3$ and [P] Dipp {CH(C*H*$_3$)$_2$}), 1.58 (d, , $^{3}J_{H–H}$ = 6.5 Hz, 3H, Dipp {CH(C*H*$_3$)$_2$}), 1.49 (d, $^{3}J_{H–H}$ = 6.8 Hz, 3H, Dipp {CH(C*H*$_3$)$_2$}), 1.32 (overlapping d, $^{3}J_{H–H}$ = 6.8 Hz, 6H, Dipp {CH(C*H*$_3$)$_2$}), 1.16 (overlapping d, *J*= 6.7 Hz, 9H), 0.99 (d, $^{3}J_{H–H}$ = 6.8 Hz, 3H, Dipp {CH(C*H*$_3$)$_2$}), 0.83 (d, *J*=6.6, 3H, Dipp {CH(C*H*$_3$)$_2$}). **^{13}C{^{1}H} NMR** (126 MHz, C_6D_6) δ = 170.87 (Nacnac {N*C*(Me)R}), 169.62 (Nacnac {N*C*(Me)R'}), 168.14 ({NC(H)R}), 150.39 (Ar*C*), 147.71 (Ar*C*), 146.86 (Ar*C*), 145.05 (d, $^{2}J_{C–P}$ = 13.7 Hz, *ipso* Ar*C*N(P)R), 144.76 (Ar*C*), 144.48 (Ar*C*), 143.07 (Ar*C*), 142.43 (Ar*C*), 140.91 (Ar*C*), 137.90 (Ar*C*), 136.46 (Ar*C*), 127.49 (Ar*C*), 126.08 (Ar*C*), 125.16 (Ar*C*), 124.97 (Ar*C*), 124.41 (Ar*C*), 124.08 (Ar*C*), 123.68 (Ar*C*), 123.29 (Ar*C*), 121.85 (Ar*C*), 98.19 (Nacnac backbone methine), 61.57 (Ga*C*(H)(N)C), 30.30 (Dipp {*C*H(CH_3)$_2$}), 29.54

(Dipp {*C*H(CH_3)$_2$}), 29.23 (Dipp {*C*H(CH_3)$_2$}), 28.72 (Dipp {*C*H(CH_3)$_2$}), 28.31 (Dipp {*C*H(CH_3)$_2$}), 28.29 (Dipp {*C*H(CH_3)$_2$}), 28.04 (Dipp {*C*H(CH_3)$_2$}), 27.83 (Dipp {*C*H(CH_3)$_2$}), 27.73 (Dipp {*C*H(CH_3)$_2$}), 27.70 (Dipp {CH(CH$_3$)$_2$}), 27.50 (Dipp {CH(CH$_3$)$_2$}), 26.43 (Dipp {CH(CH$_3$)$_2$}), 26.16 (Dipp {CH(CH$_3$)$_2$}), 25.78 (Dipp {CH(CH$_3$)$_2$}), 25.68 (Dipp {CH(CH$_3$)$_2$}), 25.42 (Dipp {CH(CH$_3$)$_2$}), 25.24 (Nacnac methyl), 24.92 (Dipp {CH(CH$_3$)$_2$}), 24.54 (Nacnac methyl), 24.29 (Dipp {CH(CH$_3$)$_2$}), 23.47 (Dipp {CH(CH$_3$)$_2$}), 23.31 (Dipp {CH(CH$_3$)$_2$}), 23.28 (Dipp {CH(CH$_3$)$_2$}), 23.25 (Dipp {CH(CH$_3$)$_2$}), 22.22 (Dipp {CH(CH$_3$)$_2$}), 21.78 (Dipp {CH(CH$_3$)$_2$}). **^{31}P{^{1}H} NMR** (162 MHz, C_6D_6) δ = 510.2 (d, $^1J_{P-P}$ = 575 Hz, phosphanyl), 122.7 (d, $^1J_{P-P}$ = 575 Hz, phosphinidene).

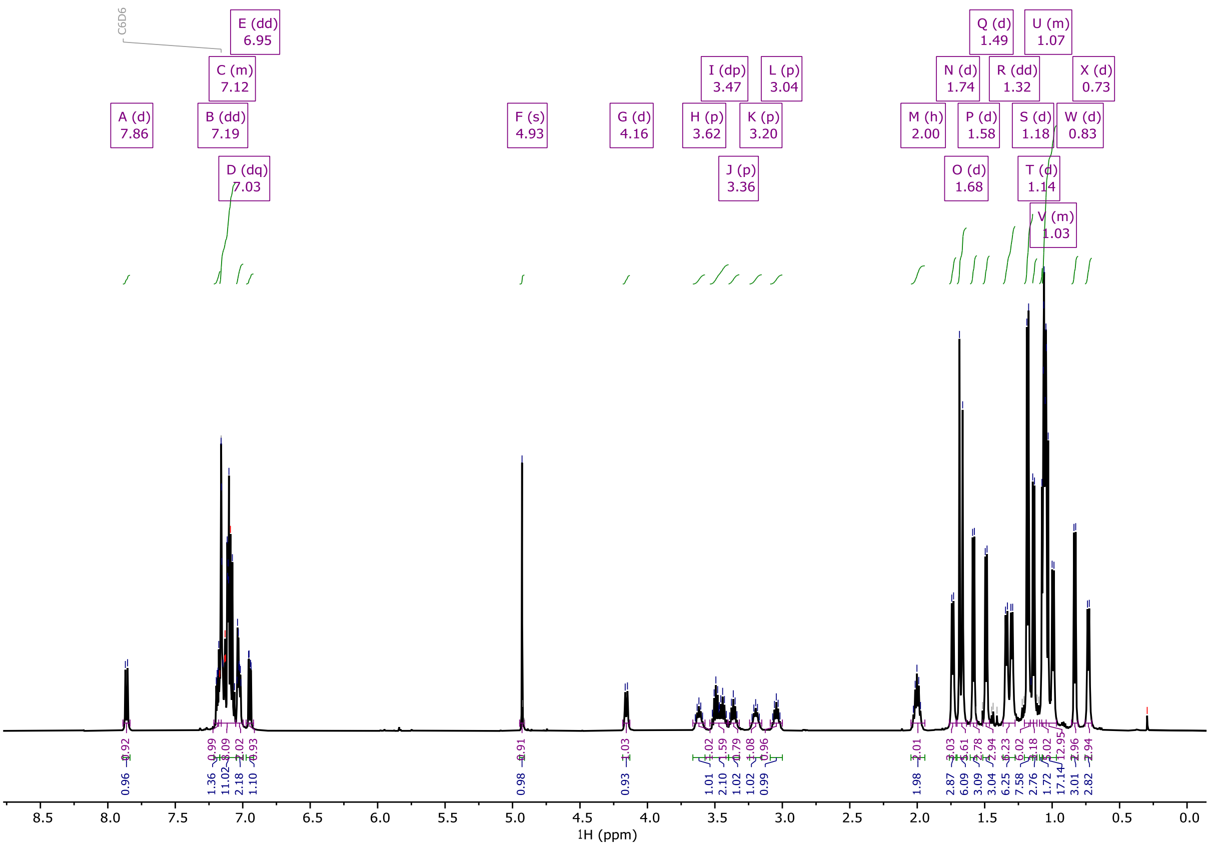

Figure S4. Room temperature ^{1}H NMR spectrum of **2** in C_6D_6.

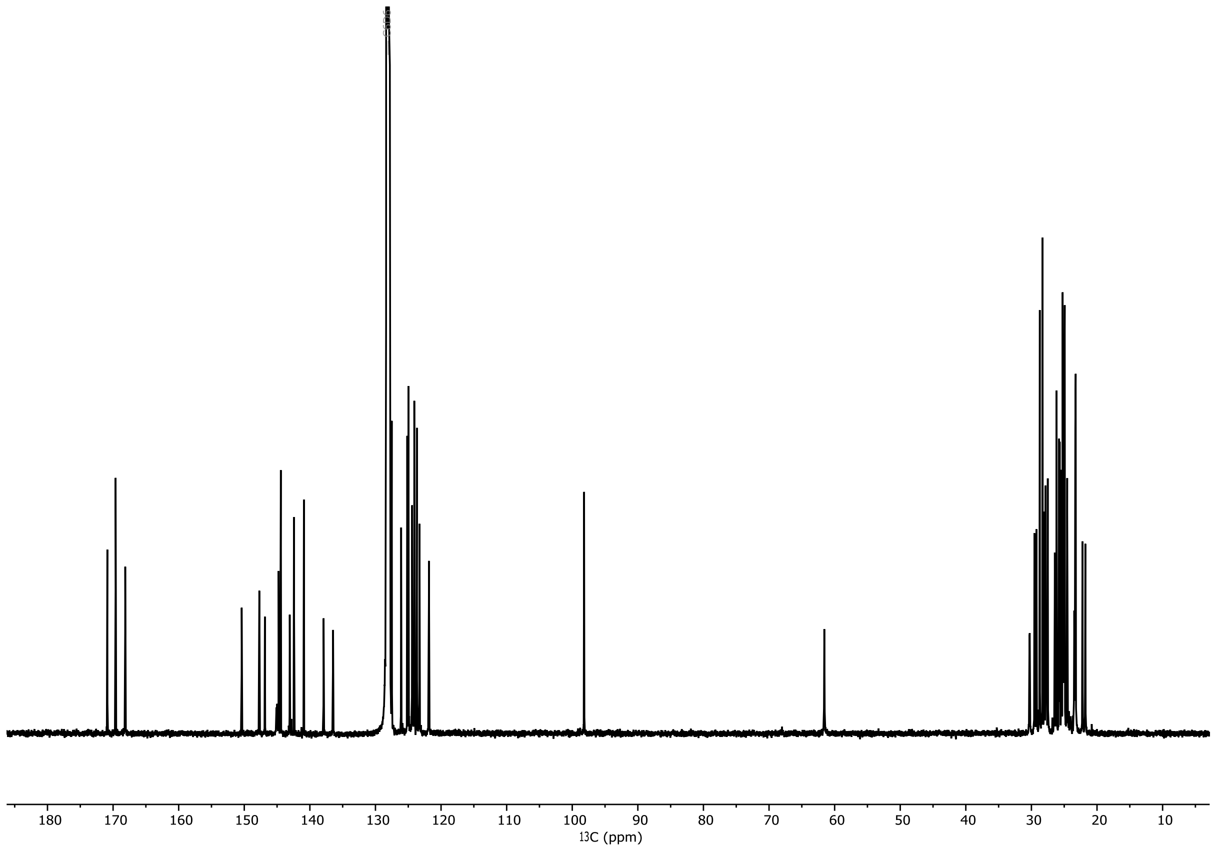

Figure S5. Room temperature ^{13}C NMR spectrum of **2** in C_6D_6.

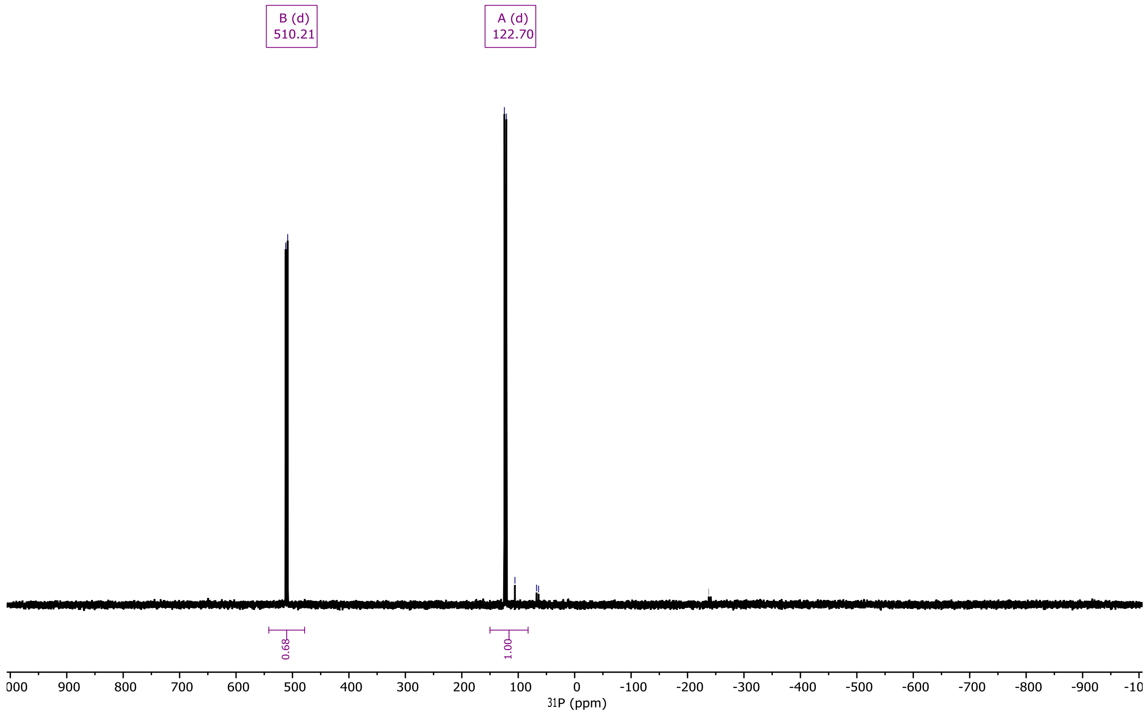

Figure S6. Room temperature $^{31}P\{^{1}H\}$ NMR spectrum of **2** in C_6D_6.

1.2.3. Synthesis of [SP]PGa(NacNac) (**1b**)

[(NacNac)Ga] (35 mg, 0.072 mmol) and a small excess of phosphanylphosphaketene [SP]PCO (40.4 mg, 0.086 mmol; 1.2 eq) were dissolved in toluene (2 mL). Immediate effervescence was observed, accompanied by a darkening of the solution from yellow to red. Complete consumption of both starting materials was confirmed by ^{1}H and ^{31}P NMR spectroscopy and conversion to **1b** can be seen to be quantitative. **1b** is stable in solution for at least 1 week and is highly sensitive to oxygen and moisture. Removal of the solvent and recrystallisation from hexane resulted in analytically pure red crystals suitable for X-ray diffraction (33 mg, 50 %). The thermal stability was assessed by heating a solution of **1b** in toluene to 80°C overnight, no change to the ^{31}P NMR spectrum was observed. The NMR sample used for the collection of spectra shown in Figures S7−S9 was left during the COVID19 departmental lockdown (11 weeks). Upon return small amounts of decomposition could be observed (Figures S10 and S11).

CHN calc'd for $C_{55}H_{79}GaN_4P_2$: C, 71.19; H, 8.58; N, 6.04. Found: C, 71.23; H, 8.63; N, 6.01. **^{1}H NMR** (400 MHz, C_6D_6): δ (ppm) 7.27–7.18 (m, 8H; ArC*H*), 7.05 (d, $^{3}J_{H–H}$ = 7.7 Hz, 4H; ArC*H*), 4.84 (s, 1H; NacNac γ-H), 4.34 (d sept, $^{3}J_{H–H}$ = 7.0 Hz, $^{5}J_{H–P}$ = 3.4 Hz, 2H; [SP] Dipp {C*H*(CH$_3$)$_2$}), 4.07–3.95 (m, 2H; {(NC*H*$_2$)$_2$}), 3.49 (d sept, $^{3}J_{H–H}$ = 7.3 Hz, $^{5}J_{H–P}$ = 3.5 Hz, 2H; [SP] Dipp {C*H*(CH$_3$)$_2$}), 3.29–3.09 (m, 2H; {(NC*H*$_2$)$_2$}), 2.90 (sept, $^{3}J_{H–H}$ = 6.8 Hz, 4H, NacNac Dipp {C*H*(CH$_3$)$_2$}), 1.43–1.36 (m, 12H, NacNac C*H*$_3$ and [SP] Dipp {CH(C*H*$_3$)$_2$}), 1.33 (d, $^{3}J_{H–H}$ = 6.8 Hz, 6H; [SP] Dipp {CH(C*H*$_3$)$_2$}), 1.28 (d, $^{3}J_{H–H}$ = 6.8 Hz, 6H; [SP] Dipp {CH(C*H*$_3$)$_2$}), 1.25 (d, $^{3}J_{H–H}$ = 6.8 Hz, 6H; [SP] Dipp {CH(C*H*$_3$)$_2$}), 1.03 (d, $^{3}J_{H–H}$ = 6.8 Hz, 12H; NacNac {CH(C*H*$_3$)$_2$}), 1.00 (d, $^{3}J_{H–H}$ = 6.8 Hz, 12H; NacNac {CH(C*H*$_3$)$_2$}). **^{13}C{^{1}H} NMR** (126 MHz, C_6D_6): δ (ppm) 169.49 (Ar*C*), 150.86 (Ar*C*), 149.63 (Ar*C*), 142.84 (Ar*C*), 141.42 (d, $^{2}J_{C–P}$ = 11.8 Hz; [SP] Dipp*C*), 140.32 (Ar*C*), 126.14 (Ar*C*), 124.88 (Ar*C*), 124.50 (Ar*C*), 123.29 (Ar*C*), 98.78 (NacNac *C*H), 54.31 (d, $^{3}J_{C–P}$ = 8.6 Hz; NacNac *C*), 28.94 (d, $^{3}J_{C–}$

$_{P}$ = 7.7 Hz; {(NCH_2)$_2$}), 28.89 (Dipp {CH(CH_3)$_2$}) 28.43 (d, $^3J_{C–P}$ = 7.7 Hz; {(NCH_2)$_2$}), 26.84 ({CH(CH_3)$_2$}), 26.58 ({CH(CH_3)$_2$}), 25.66 (d, $^4J_{C–P}$ = 3.8 Hz; NacNac CH_3), 24.86 (Dipp {CH(CH_3)$_2$}), 24.29 (Dipp{CH(CH_3)$_2$}), 24.11 (br s, Dipp {CH(CH_3)$_2$}), 23.48 (Dipp {CH(CH_3)$_2$}). **$^{31}P\{^1H\}$ NMR** (162 MHz, C_6D_6): δ (ppm) 157.82 (d, $^1J_{P–P}$ = 345.8 Hz, phosphanyl), –61.27 (d, $^1J_{P–P}$ = 345.8 Hz, phosphinidene).

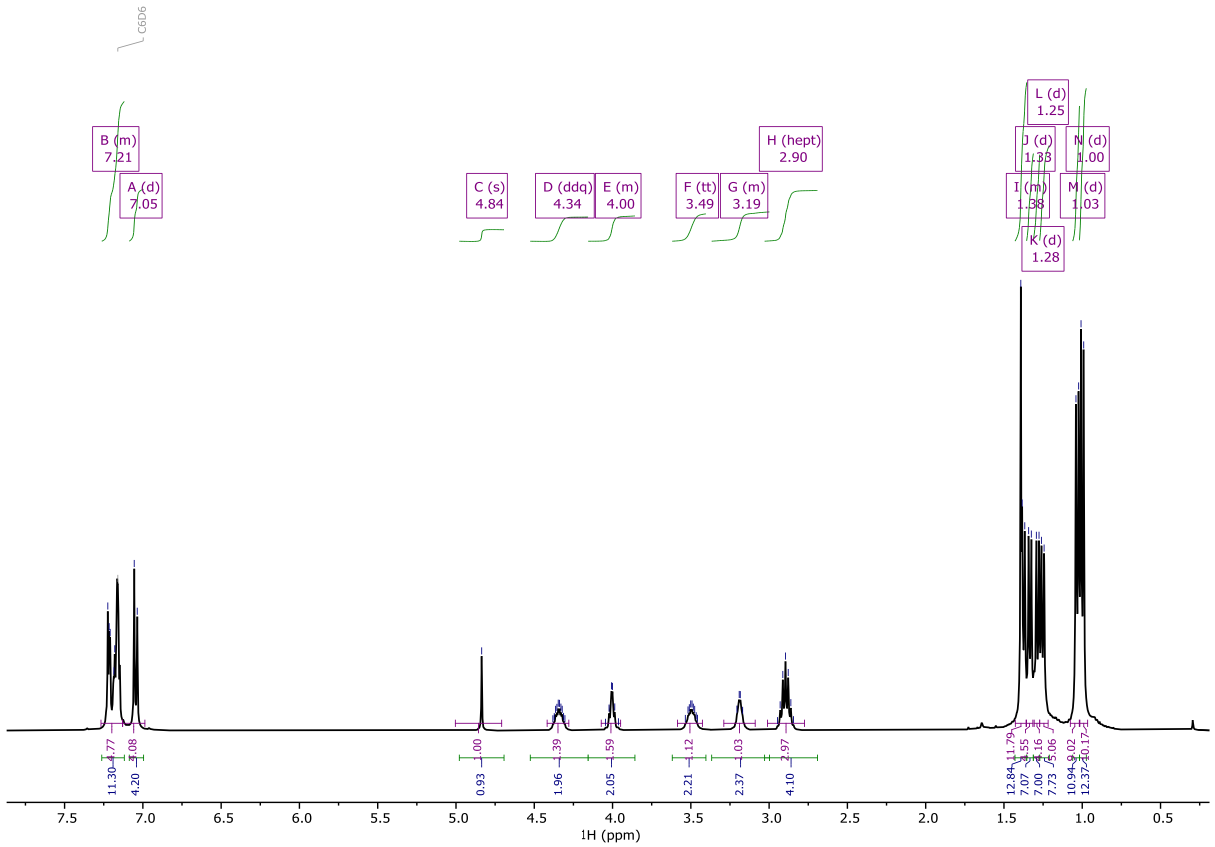

Figure S7. Room temperature ^{1}H NMR of **1b** in C_6D_6.

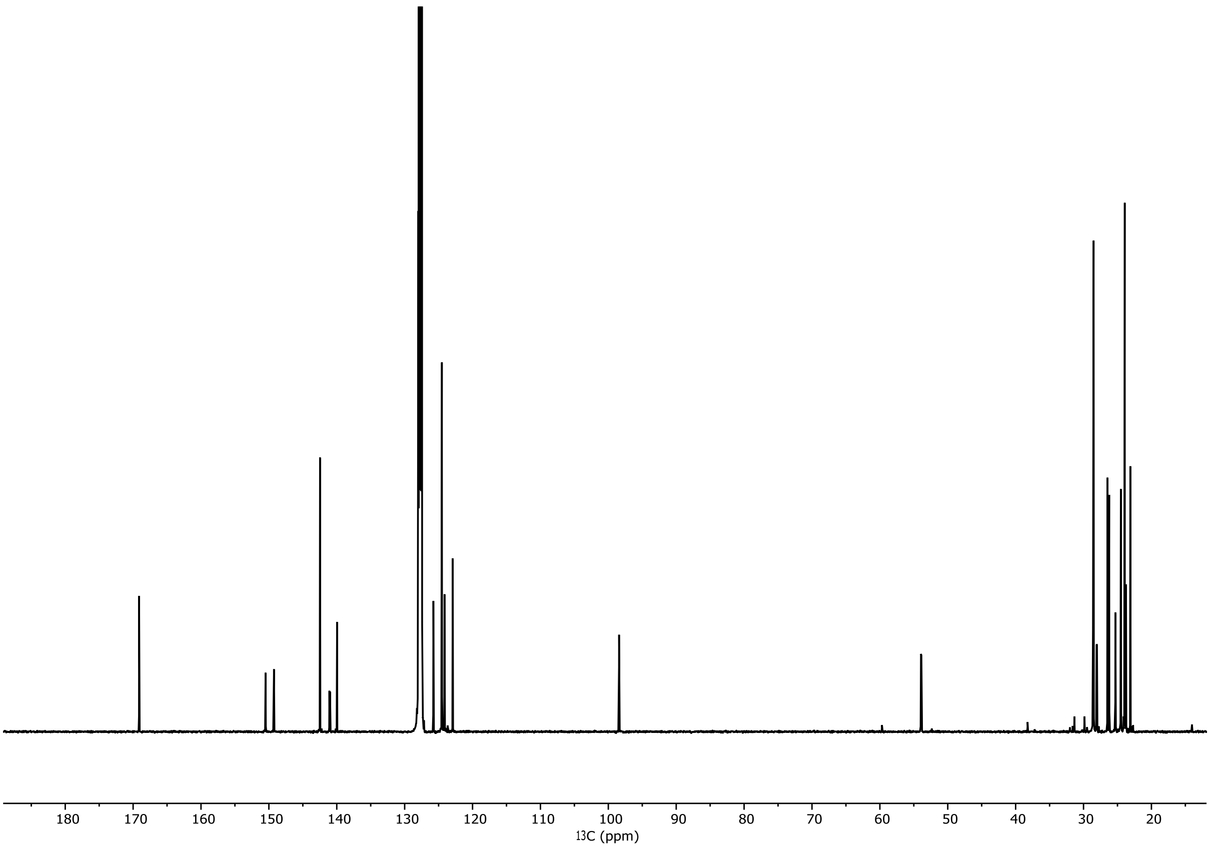

Figure S8. Room temperature $^{13}C\{^{1}H\}$ NMR of **1b** in C_6D_6.

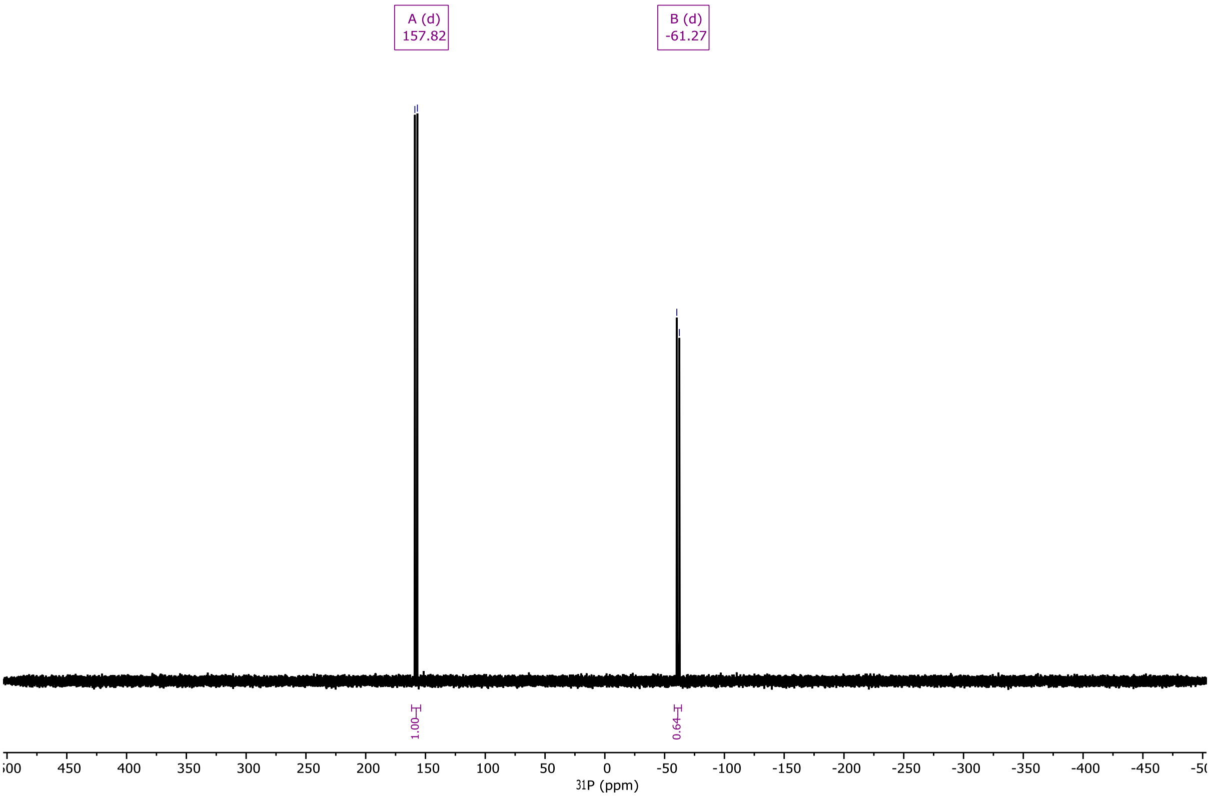

Figure S9. Room temperature $^{31}P\{^{1}H\}$ NMR of **1b** in C_6D_6.

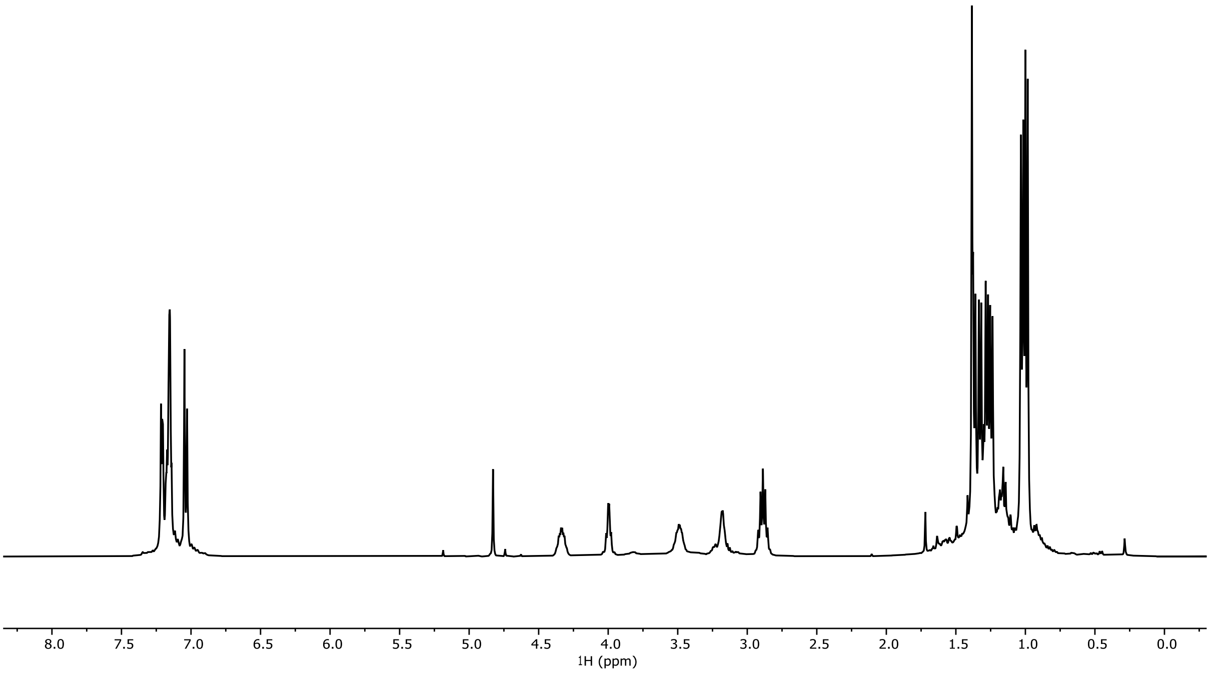

Figure S10. Room temperature ^{1}H NMR of **1b** in C_6D_6 after 11 weeks standing in solution.

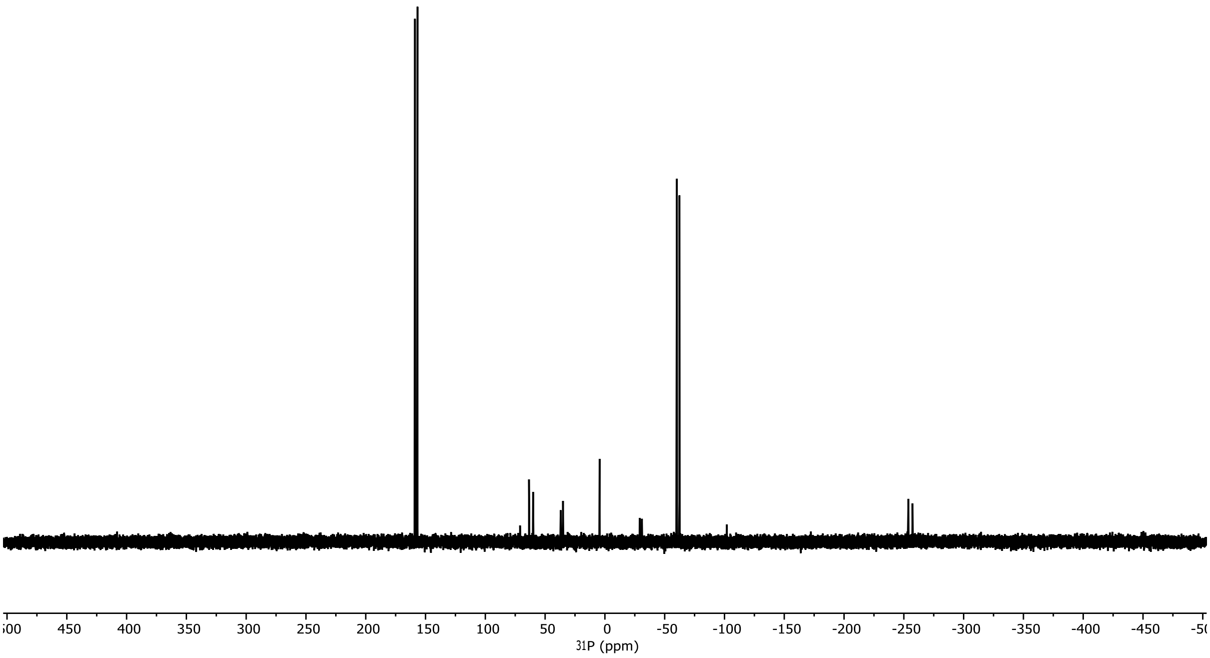

Figure S11. Room temperature $^{31}P\{^{1}H\}$ NMR of **1b** in C_6D_6 after 11 weeks standing in solution.

1.2.4. Synthesis of [SP]P(H)Ga(H)(NacNac) (**3**)

A toluene (0.5 mL) solution of **1b** (40 mg, 0.043 mmol) was prepared *in situ* as described in section 1.4. The solution was added to a gas-tight NMR tube and degassed using the freeze-pump-thaw method. The headspace was replaced by 2 bar of H_2. The solution immediately lightened in colour, turning from red to orange. Conversion to a new product was quantitative by ^{1}H and ^{31}P NMR spectroscopy. Removal of the solvent produced a light-yellow solid which was insoluble in hexane or pentane. Slow evaporation of a toluene solution at room temperature yielded colourless crystals suitable for X-ray diffraction. Placing the crystallised mixture into the freezer induced precipitation of **3** as a microcrystalline powder (27 mg, 67 %).

CHN calc'd for $C_{55}H_{79}GaN_4P_2$: C, 71.19; H, 8.58; N, 6.04. Found: C, 70.63; H, 8.10; N, 5.89. **^{1}H NMR** (500 MHz, C_6D_6): δ (ppm) 8.93 (dd, $^1J_{H–P}$ = 456.9 Hz, $^2J_{H–P}$ = 10.0 Hz, 1H; [SP]*H*), 7.19 (t, $^3J_{H–H}$ = 7.6 Hz, 2H; *para*-ArC*H*), 7.10 (m, 4H; ArC*H*), 7.04 – 6.92 (m, 6H; ArC*H*), 5.80 (br s, 1H, [Ga]*H*), 4.72 (s, 1H; NacNac γ-H), 3.84 (sept, $^3J_{H–H}$ = 6.8 Hz, 2H; {C*H*(CH_3)$_2$}), 3.38 (sept, $^3J_{H–H}$ = 6.9 Hz, 2H; {C*H*(CH_3)$_2$}), 3.33–3.11 (m, 8H; [Ga] {C*H*(CH_3)$_2$} and [SP]{(NC*H*$_2$)$_2$}), 1.39 (s, 6H; NacNac C*H*$_3$), 1.38–0.79 (m, 58H; all Dipp {CH(C*H*$_3$)$_2$}). **^{13}C{^{1}H} NMR** (126 MHz, C_6D_6) δ = 168.44 (Ar*C*), 150.46 (Ar*C*), 149.42 (Ar*C*), 144.28 (Ar*C*), 143.60 (Ar*C*), 142.98 (Ar*C*), 136.80 (d, $^2J_{C–P}$ = 5.2 Hz; Ar*C*), 126.75 (Ar*C*), 124.60 (Ar*C*), 124.04 (Ar*C*), 123.97 (Ar*C*), 123.91 (Ar*C*), 95.71 (NacNac *C*H), 60.06 (unknown/impurity), 51.05 (d, $^3J_{C–P}$ = 5.7 Hz; NacNac *C*), 38.61 (Dipp{CH(CH_3)$_2$}), 32.37 (Dipp{CH(CH_3)$_2$}), 31.71 (Dipp{CH(CH_3)$_2$}), 30.23 (NacNac *C*H$_3$), 26.57 (Dipp{CH(CH_3)$_2$}), 26.46 (Dipp{CH(CH_3)$_2$}), 25.29 (Dipp{CH(CH_3)$_2$}), 25.15 (Dipp{CH(CH_3)$_2$}), 24.92 (Dipp{CH(CH_3)$_2$}), 24.50 (dd, *J* =22.2 Hz, 6.9 Hz, unknown), 24.09 (Dipp{CH(CH_3)$_2$}), 23.78 (Dipp{CH(CH_3)$_2$}), 23.14 (Dipp{CH(CH_3)$_2$}), 14.39 (Dipp{CH(CH_3)$_2$}). **^{31}P{^{1}H} NMR** (162 MHz, C_6D_6) δ = 66.96 (d, $^1J_{P–P}$ = 578.4 Hz; [S*P*]H), –248.52 (d, $^1J_{P–P}$ = 578.4 Hz, phosphinidene

P).**³¹P NMR** (162 MHz, C_6D_6) δ = 66.96 (dd, $^1J_{P–P}$ = 577.8 Hz, $^1J_{P–H}$ = 457.1 Hz; [S*P*]H), –248.67 (d, $^1J_{P–P}$ = 578.4 Hz, phosphinidene *P*).

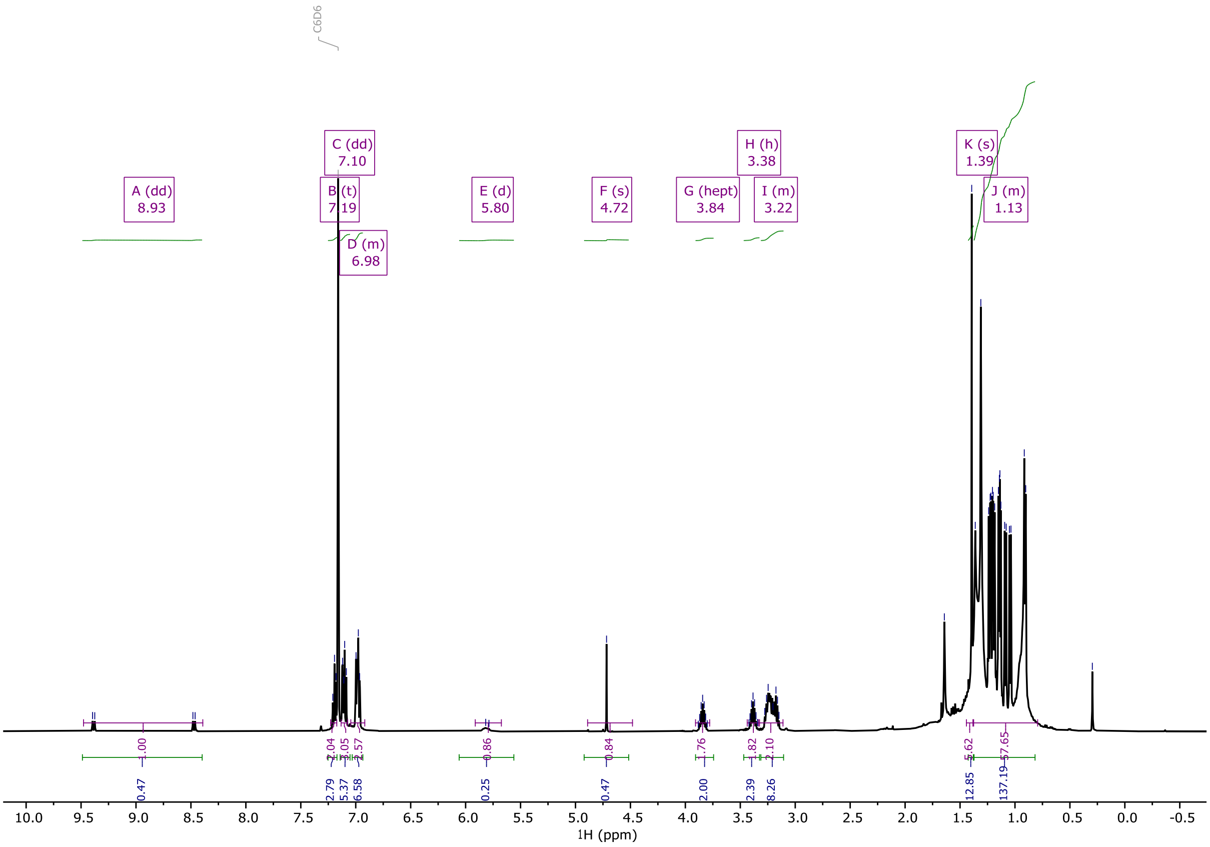

Figure S12. Room temperature ¹H NMR of **3** in C_6D_6.

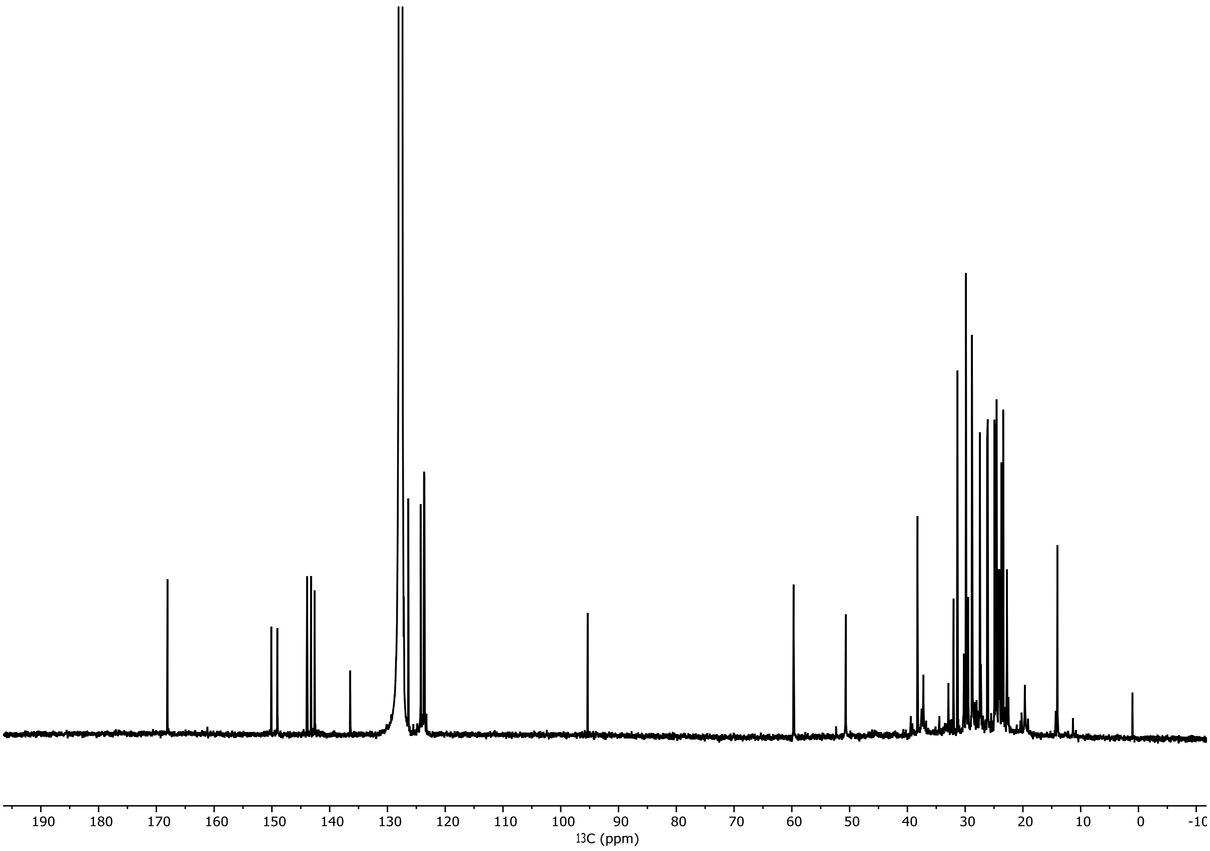

Figure S13. Room temperature ^{31}C{^{1}H} NMR of **3** in C_6D_6.

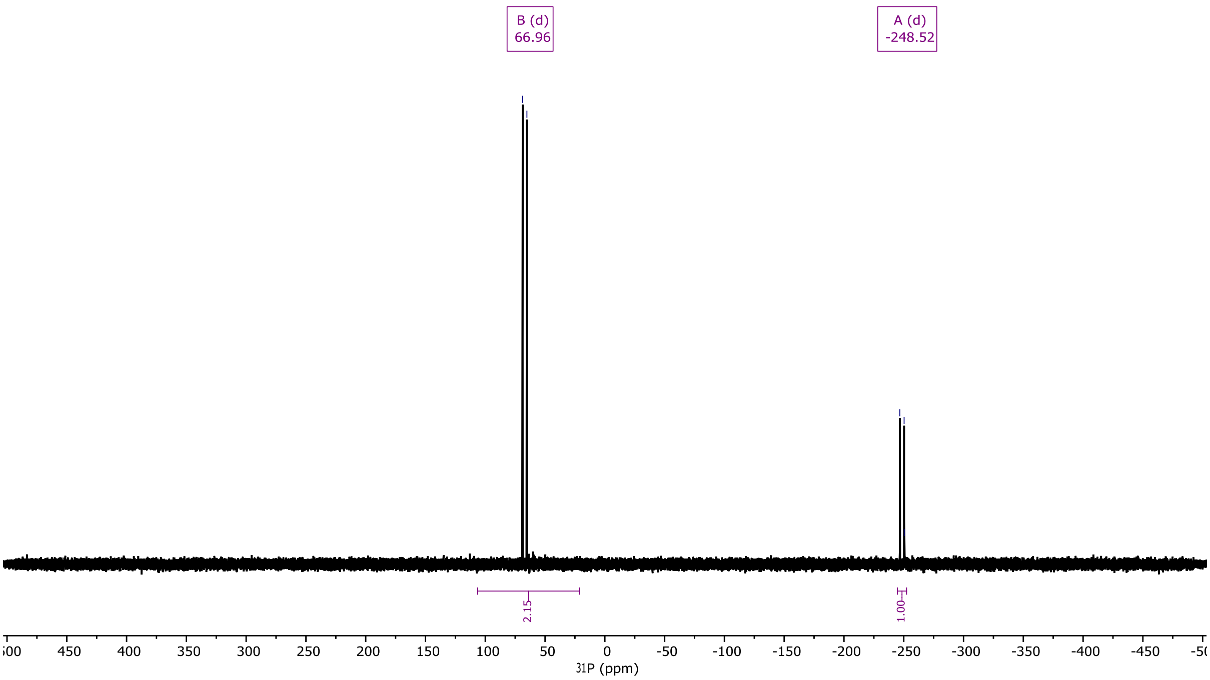

Figure S14. Room temperature ^{31}P{^{1}H} NMR spectrum of **3** in C_6D_6.

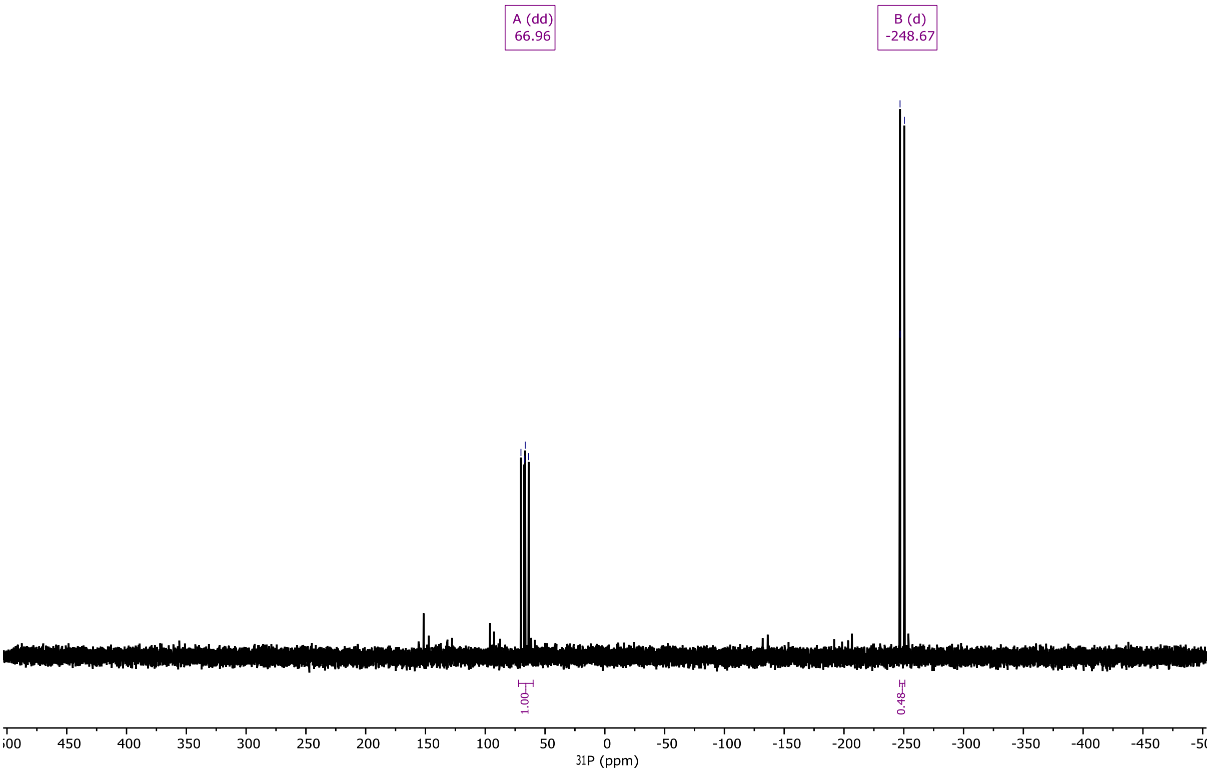

Figure S15. Room temperature ^{31}P NMR spectrum of **3** in C_6D_6.

1.2.5. Activation of CO_2 by **1b** to yield **4**.

A toluene (0.5 mL) solution of **1b** (40 mg, 0.043 mmol) was prepared *in situ* as described in section 1.4. The solution was added to an air-tight NMR tube and degassed using the freeze-pump-thaw method. The headspace was replaced by 2 bar of CO_2. The solution immediately lightened in colour, turning from red to orange. Conversion to a new product was quantitative by ^{1}H and ^{31}P NMR spectroscopy. Removal of the solvent resulted in an orange oil, which upon washing with small amounts of hexane (3 × 0.5 mL) precipitated out an analytically pure white solid (31 mg, 74%). The hexane washings were filtered and allowed to sit at room temperature for two days, forming large colourless crystals of **4** suitable for X-ray diffraction (*ca.* 5 mg).

CHN calc'd for $C_{56}H_{79}GaN_4O_2P_2$: C, 69.12; H, 8.19; N, 5.76. Found: C, 69.12; H, 8.21; N, 5.86. **^{1}H NMR** (500 MHz, C_6D_6): δ (ppm) 7.18 (d, $^{3}J_{H–H}$ = 7.6 Hz, 2H; ArC*H*), 7.14–7.01 (m, 6H; ArC*H*), 6.92 (m, 2H; ArC*H*), 6.81 (m, 2H; ArC*H*), 4.67 (s, 1H; NacNac γ-H), 3.90 (sept, $^{3}J_{H–H}$ =6.9 Hz, 2H; {C*H*(CH$_3$)$_2$}), 3.82–3.68 (m, 2H; {(NC*H*$_2$)$_2$}), 3.60 (sept, $^{3}J_{H–H}$ = 6.8 Hz, 2H; {C*H*(CH$_3$)$_2$}), 3.37 (sept, $^{3}J_{H–H}$ =6.9 Hz, 2H; {C*H*(CH$_3$)$_2$}), 3.23–3.10 (m, 2H; {(NC*H*$_2$)$_2$}), 3.04 (sept, $^{3}J_{H–H}$ = 6.8 Hz, 2H; {C*H*(CH$_3$)$_2$}), 1.69 (d, $^{3}J_{H–H}$ = 6.8 Hz, 6H; {CH(C*H*$_3$)$_2$}), 1.60 (d, $^{3}J_{H–H}$ = 6.8 Hz, 6H; {CH(C*H*$_3$)$_2$}), 1.31 (s, 6H; NacNac C*H*$_3$), 1.23 (d, $^{3}J_{H–H}$ = 6.8 Hz, 6H; {CH(C*H*$_3$)$_2$}), 1.15 (d, $^{3}J_{H–H}$ = 6.9 Hz, 6H; {CH(C*H*$_3$)$_2$}), 1.09 (d, $^{3}J_{H–H}$ = 6.9 Hz, 6H; {CH(C*H*$_3$)$_2$}), 0.99 (d, $^{3}J_{H–H}$ = 6.6 Hz, 6H; {CH(C*H*$_3$)$_2$}), 0.67 (d, $^{3}J_{H–H}$ = 6.8 Hz, 6H; {CH(C*H*$_3$)$_2$}), 0.50 (d, $^{3}J_{H–H}$ = 6.8 Hz, 6H; {CH(C*H*$_3$)$_2$}). **^{13}C{^{1}H} NMR** (126 MHz, C_6D_6): δ (ppm) 174.35 (dd, $^{1}J_{C–P}$ = 100.6, $^{2}J_{C–P}$ = 11.0 Hz; O*C*O), 170.09 (Ar*C*), 151.77 (Ar*C*), 149.72 (Ar*C*), 145.45 (Ar*C*), 144.96 (Ar*C*), 144.08 (Ar*C*), 140.57 (Ar*C*), 137.10 (d, $^{2}J_{C–P}$ = 6.8 Hz, Ar*C*), 125.07 (Ar*C*), 124.64 (Ar*C*), 124.61 (Ar*C*), 124.49 (Ar*C*), 96.75 (NacNac *C*H), 51.09 ($^{3}J_{C–P}$ = 4.8 Hz; NacNac *C*), 31.97 (Dipp{CH(*C*H$_3$)$_2$}), 29.33 (d, $^{3}J_{C–P}$ = 4.2 Hz Dipp{CH(*C*H$_3$)$_2$}), 8.74 (Dipp{CH(*C*H$_3$)$_2$}), 28.19 (d, $^{4}J_{C–P}$ = 2.5 Hz, nacnac *C*H$_3$)), 27.84 (Dipp{CH(CH$_3$)$_2$}), 27.39 (Dipp{CH(CH$_3$)$_2$}), 26.85 (Dipp{CH(CH$_3$)$_2$}), 26.81

(Dipp{CH(CH_3)$_2$}), 25.88 (Dipp{CH(CH_3)$_2$}), 25.36 (Dipp{CH(CH_3)$_2$}), 25.07 (Dipp{CH(CH_3)$_2$}), 24.70 (Dipp{CH(CH_3)$_2$}), 24.32 (Dipp{CH(CH_3)$_2$}), 23.97 (Dipp{CH(CH_3)$_2$}), 23.91 (Dipp{CH(CH_3)$_2$}), 23.06 (Dipp {CH(CH_3)$_2$}), 14.37 (unknown). **^{31}P{^{1}H} NMR** (162 MHz, C_6D_6) δ = 80.67 (d, $^1J_{P–P}$ = 588.4 Hz), –290.95 (d, $^1J_{P–P}$ = 588.4 Hz).

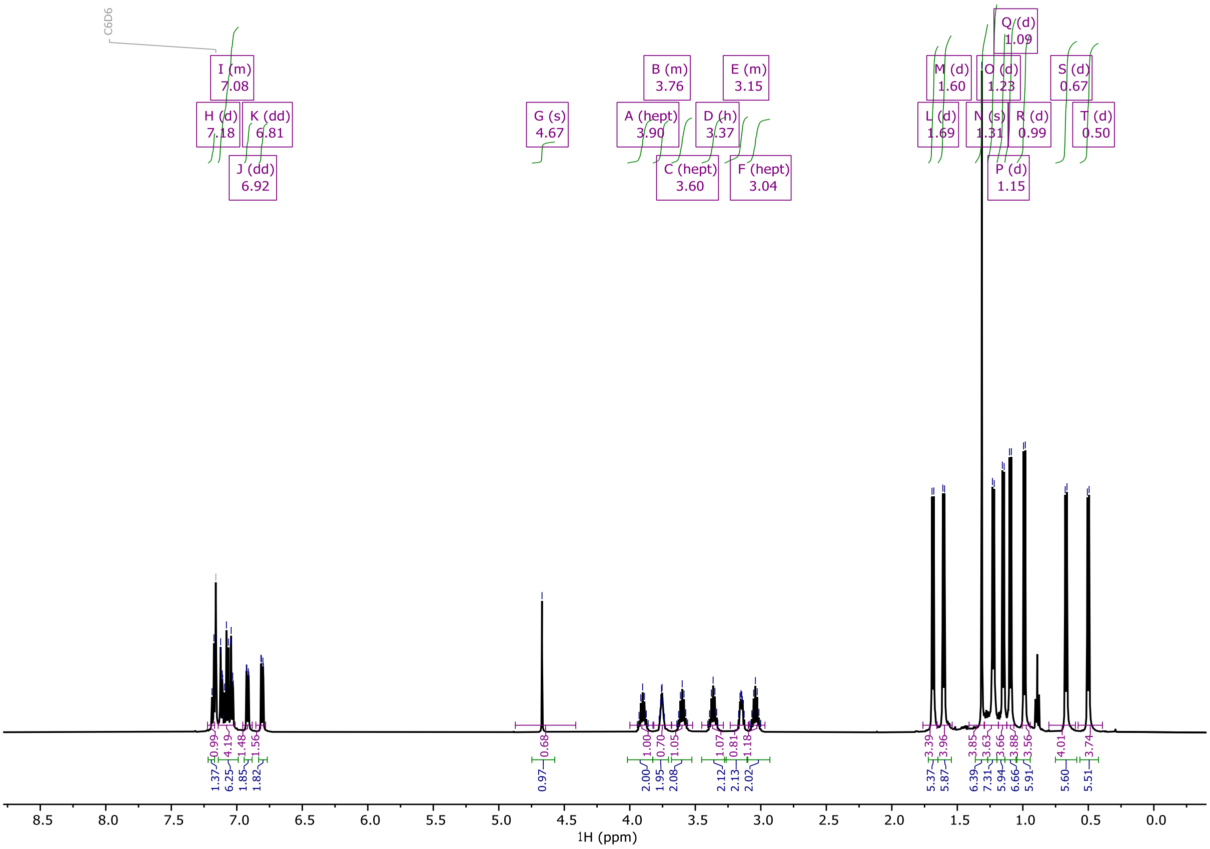

Figure S16. Room temperature ^{1}H NMR spectrum of **4** in C_6D_6.

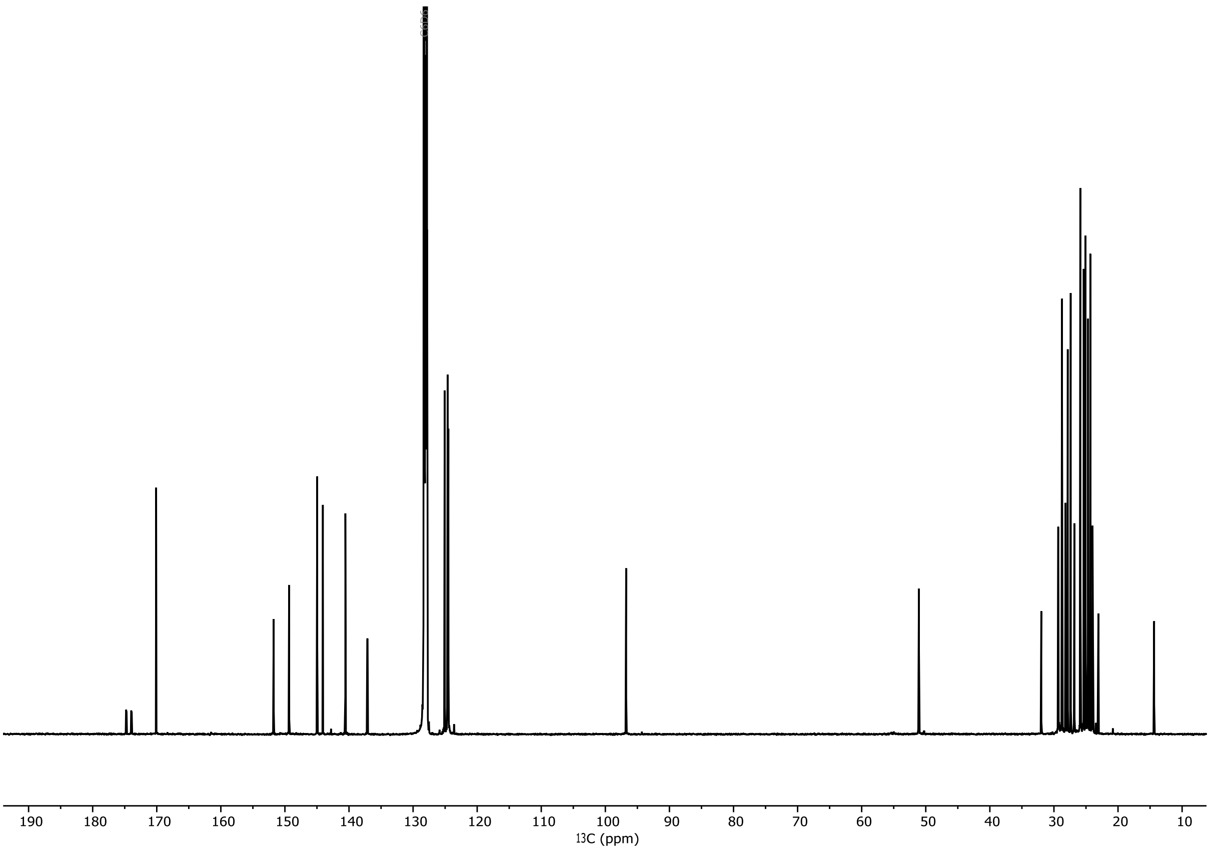

Figure S17. Room temperature $^{13}C\{^{1}H\}$ NMR spectrum of **4** in C_6D_6.

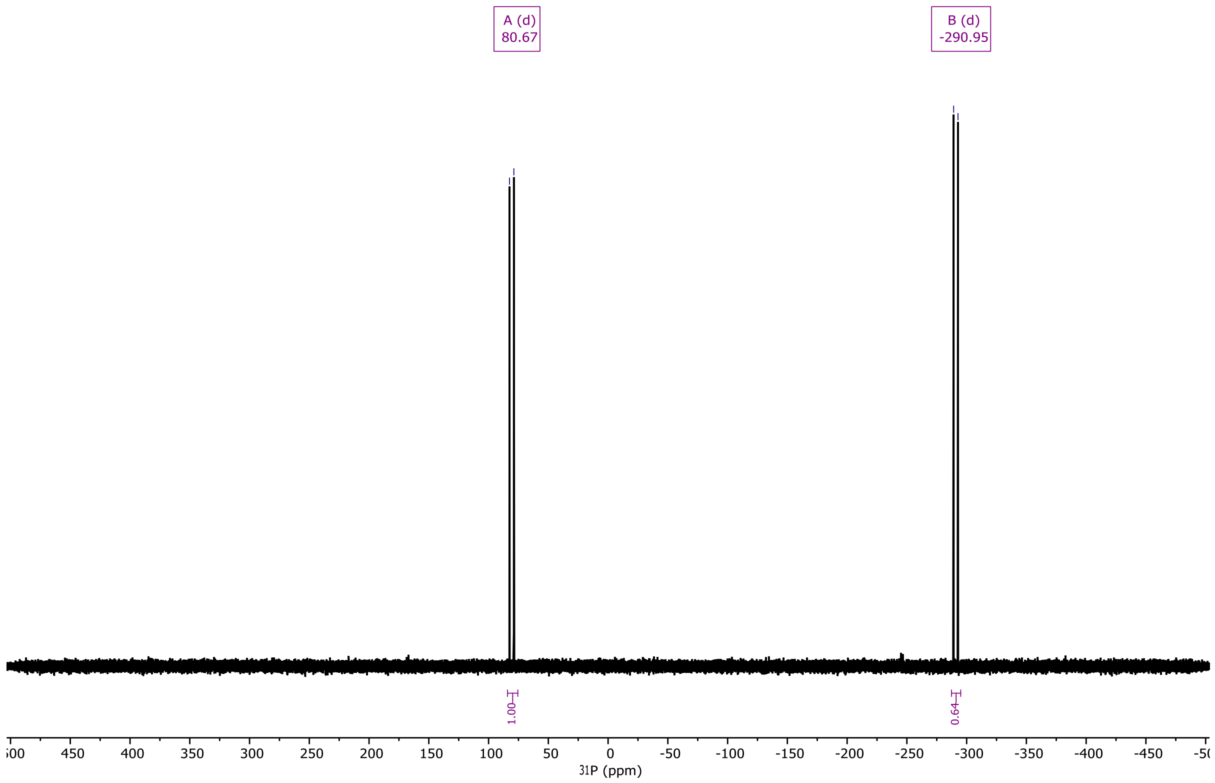

Figure S18. Room temperature $^{31}P\{^{1}H\}$ spectrum of **4** in C_6D_6.

1.2.6. Variable temperature NMR experiments

To probe the magnitude of the π-contribution to the Ga=P bond VT-NMR was performed on a solution of **1b**. The methine proton of the diisopropylphenyl groups is expected to split into two distinct resonances upon hindered bond rotation from which the energy can be calculated.[4] Cooling to –80°C resulted in broadening of the ^{1}H NMR resonance, however no resolution was observed implying a contribution of less than 10.2 kcal/mol. The related PhP(GaTrip$_2$)$_2$ (Trip = 2,4,6-*i*Pr$_3$C$_6$H$_2$) displays resolution of 79 Hz at –95°C for a calculated π-contribution of 10.2 kcal/mol for the dynamic process.[7] The authors do not report the solvent used for this experiment (presumably toluene). We were hesitant to further lower the temperature due to the possibility of the solution freezing.

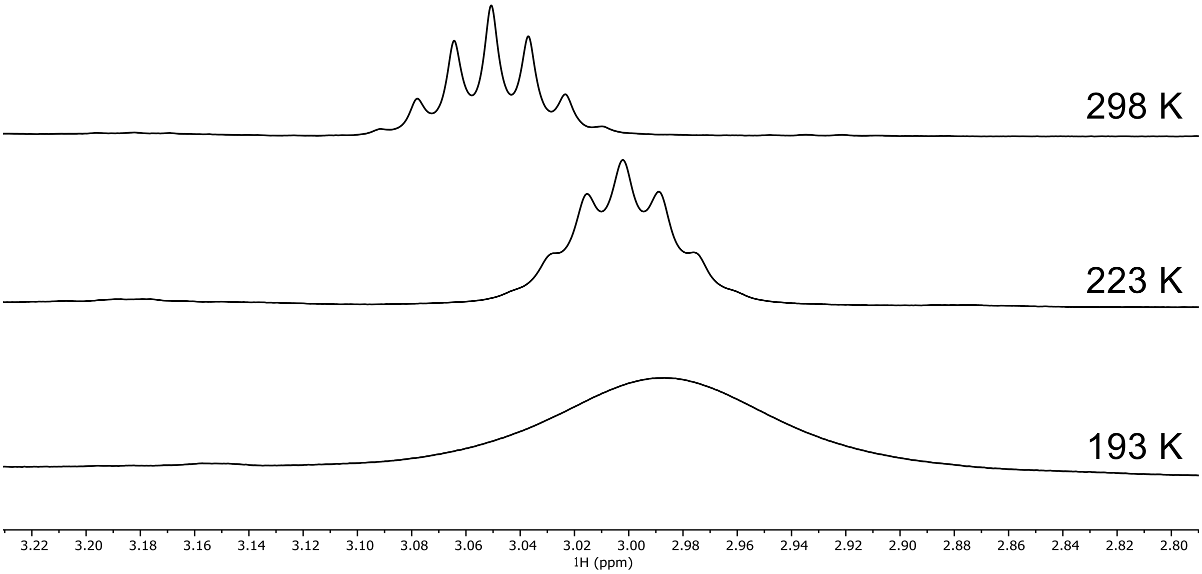

Figure S19. VT-NMR experiment on a solution of **1b** in d$_8$-toluene displaying the Nacnac isopropyl methine region.

2. Single crystal X-ray diffraction data

Single-crystal X-ray diffraction data were collected using an Oxford Diffraction Supernova dual-source diffractometer equipped with a 135 mm Atlas CCD area detector. Crystals were selected under Paratone-N oil, mounted on micromount loops and quench-cooled using an Oxford Cryosystems open flow N_2 cooling device. Data were collected at 150 K using mirror monochromated Cu K_α radiation ($\lambda = 1.5418$ Å; Oxford Diffraction Supernova) and processed using the CrysAlisPro package, including unit cell parameter refinement and inter-frame scaling (which was carried out using SCALE3 ABSPACK within CrysAlisPro).[5] Equivalent reflections were merged and diffraction patterns processed with the CrysAlisPro suite. Structures were subsequently solved using direct methods and refined on F^2 using the SHELXL package.[6]

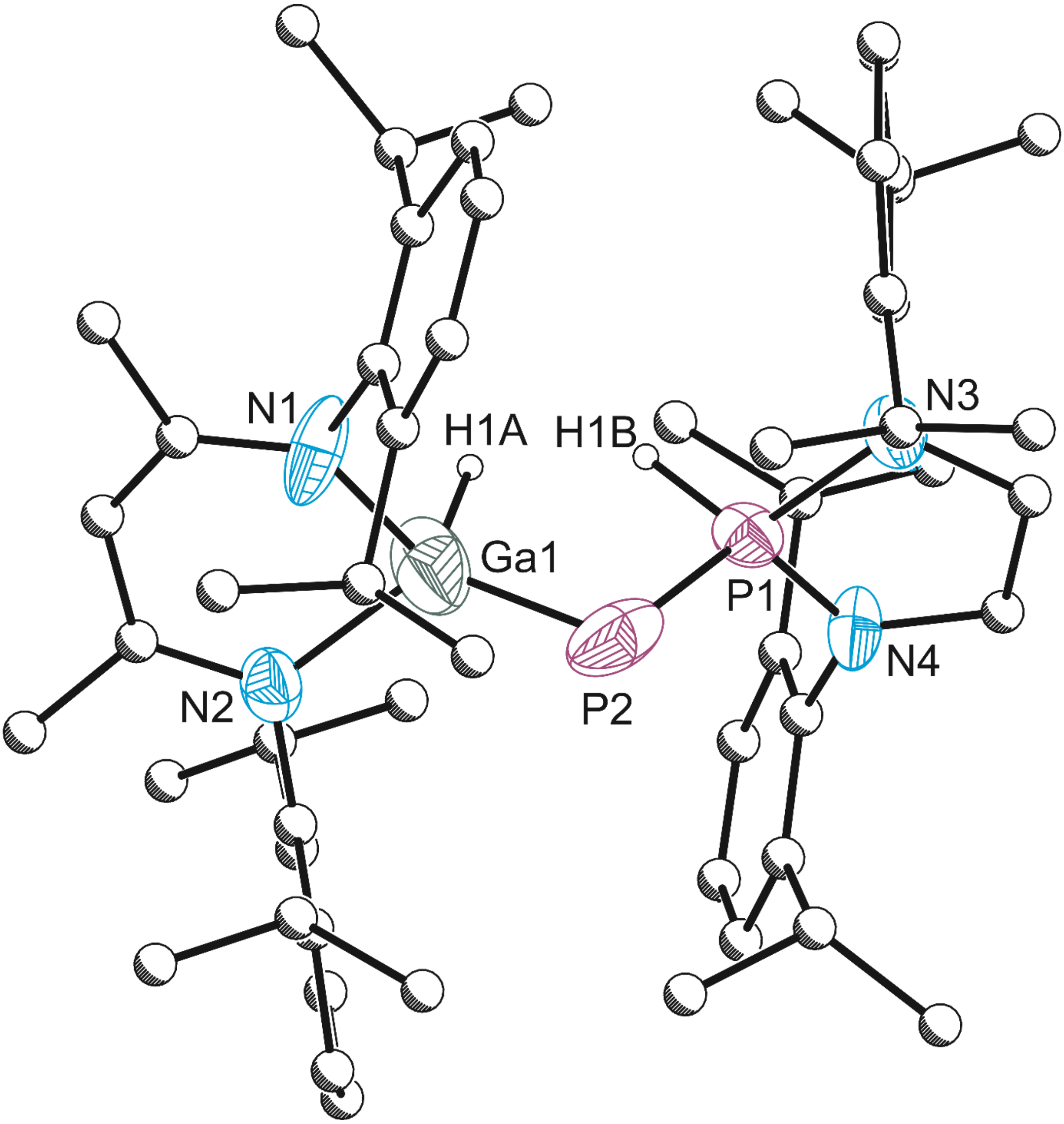

Figure S20. Molecular structure of **3**. Ellipsoids set at 50% probability. Significant positional disorder results in poor structure solution and refinement, however allows for the elucidation of the connectivity of **3**.

Table S1. Selected X-ray data collection and refinement parameters for **1a**·hex, **1b** and **2**·0.5hex.

	**1a**·hex	**1b**	**2**·0.5hex
Formula	$C_{61}H_{91}GaN_4P_2$	$C_{55}H_{79}GaN_4P_2$	$C_{58}H_{84}GaN_4P_2$
CCDC	2008786	2008787	2008788
Fw [g mol^{-1}]	1012.03	927.88	968.95
Crystal system	monoclinic	monoclinic	monoclinic
Space group	$P2_1/c$	$P2_1/c$	$P2_1/c$
a (Å)	20.7083(4)	21.1076(2)	15.1634(3)
b (Å)	12.7880(2)	13.75640(10)	16.9614(3)
c (Å)	25.0002(5)	20.8759(2)	21.8303(3)
α (°)	90	90	90
β (°)	113.959(2)	118.0770(10)	99.407(2)
γ (°)	90	90	90
V (Å^3)	6050.0(2)	5348.26(9)	5539.08(17)
Z	4	4	4
Radiation, λ (Å)	Cu Kα, 1.54184	Cu Kα, 1.54184	Cu Kα, 1.54184
Temp (K)	150(2)	150(2)	150(2)
ρ_{calc} (g cm^{-3})	1.111	1.152	1.162
μ (mm^{-1})	1.401	1.544	1.510
Reflections collected	52884	90489	37459
Independent reflections	12699	11172	11457
Parameters	631	577	605
R(int)	0.0435	0.0307	0.0305
R1/wR2,[a] I ≥ 2σI (%)	4.74/13.07	2.87/7.72	4.70/11.91
R1/wR2,[a] all data (%)	6.28/14.12	3.21/7.98	6.22/13.00
GOF	1.086	1.035	0.989

$R1 = [\Sigma||F_o| - |F_c||]/\Sigma|F_o|$; $wR2 = \{[\Sigma w[(F_o)^2 - (F_c)^2]^2]/[\Sigma w(F_o^2)^2]\}^{1/2}$; $w = [\sigma^2(F_o)^2 + (AP)^2 + BP]^{-1}$, where $P = [(F_o)^2 + 2(F_c)^2]/3$ and the A and B values are 0.0701 and 3.2584 for **1a**·hex, 0.0436 and 1.5521 for **1b** and 0.0676 and 3.7516 for **2**·0.5hex.

Table S2. Selected X-ray data collection and refinement parameters for **3** and **4**·3hex.

	**3**	**4**·3hex
Formula	$C_{55}H_{81}GaN_4P_2$	$C_{74}H_{121}GaN_4O_2P_2$
CCDC	2008789	2008790
Fw [g mol^{-1}]	928.89	1230.40
Crystal system	monoclinic	monoclinic
Space group	*P*n	*C*2/m
a (Å)	13.6106(5)	18.8824(2)
b (Å)	14.7602(4)	17.68860(10)
c (Å)	14.2019(5)	20.2963(2)
α (°)	90	90
β (°)	111.221(4)	103.9980(10)
γ (°)	90	90
V (Å^3)	2659.63(17)	6577.72(11)
Z	2	4
Radiation, λ (Å)	Cu Kα, 1.54184	Cu Kα, 1.54184
Temp (K)	150(2)	150(2)
ρ_{calc} (g cm^{-3})	1.161	1.242
μ (mm^{-1})	1.552	1.398
Reflections collected	13396	37372
Independent reflections	6324	7083
Parameters	600	494
R(int)	0.0361	0.0200
R1/wR2,[a] I ≥ 2σI (%)	9.35/24.80	4.54/14.11
R1/wR2,[a] all data (%)	10.47/26.23	4.64/14.21
GOF	1.034	1.138

$R1 = [\Sigma||F_o| - |F_c||]/\Sigma|F_o|$; $wR2 = \{[\Sigma w[(F_o)^2 - (F_c)^2]^2]/[\Sigma w(F_o^2)^2\}^{1/2}$; $w = [\sigma^2(F_o)^2 + (AP)^2 + BP]^{-1}$, where $P = [(F_o)^2 + 2(F_c)^2]/3$ and the A and B values are 0.1520 and 3.9040 for **3** and 0.0858 and 7.5470 for **4**·3hex.

3. Computational details

Where available, optimisations were performed starting from the crystal structure geometry. Initial conformational optimisations were performed using a Hartree-Fock/STO-3G method as the number of isopropyl groups made convergence time consuming. For **1a** and **1b**, these structures were taken as the basis for optimisations using B3LYP/6-31g(d,p) and then finally optimisations were performed at B3LYP using Def2TZVP (Ga, P, N) and Def2SVP (C, H). The final geometry was characterised as a true minima via harmonic frequency calculations. All calculations were performed on the Gaussian16 software package, natural bond order analysis was performed using NBO version 6.0 and NMR calculations were performed with the keyword NMR. NICS calculations on **2** were performed using PBE/6-31g(d,p).

3.1. NICS Calculations

The isotropic shielding constants were computed using the protocol outlined in section 3. Dummy atoms were placed at the centroid (NICS(0)) and 1 Angstrom either side (NICS(1)), the latter of which was taken as a mean of the two values (4.0512 and 3.9894).

3.2. Natural Bond Orbital calculations

3.2.1. **1a**

```
    (Occupancy)   Bond orbital/ Coefficients/ Hybrids
 ---------------------------------------------------------------------------------
   1. (1.97051) BD ( 1)Ga   1 - P   3
               ( 40.67%)   0.6377*Ga   1 s( 84.03%)p 0.19( 15.83%)d 0.00(  0.11%)
                                                  f 0.00(  0.03%)
                                           -0.0000  0.0000 -0.0000 -0.9167 -0.0063
                                            0.0031 -0.0000 -0.0003 -0.2903 -0.0241
                                            0.0012 -0.0000 -0.0003 -0.2035 -0.0328
                                            0.0000  0.0000  0.0003  0.1696  0.0463
                                            0.0005 -0.0002  0.0070  0.0041  0.0049
                                            0.0006 -0.0218 -0.0053 -0.0039 -0.0003
                                           -0.0036 -0.0096 -0.0068  0.0004 -0.0166
                                           -0.0017  0.0015 -0.0000  0.0087  0.0037
                                            0.0002 -0.0049 -0.0041 -0.0061 -0.0005
                                            0.0128  0.0027 -0.0057
               ( 59.33%)   0.7703* P   3 s( 13.88%)p 6.14( 85.19%)d 0.07(  0.93%)
                                                  f 0.00(  0.00%)
                                           -0.0000  0.0002 -0.3725  0.0073 -0.0002
                                            0.0001  0.4394  0.0010 -0.0002  0.0007
                                            0.0001  0.5033 -0.0051  0.0003  0.0007
                                           -0.0000 -0.6368 -0.0007 -0.0005 -0.0007
                                           -0.0338 -0.0014  0.0470  0.0037  0.0679
                                           -0.0004  0.0136  0.0022 -0.0340  0.0016
                                            0.0004 -0.0009  0.0019  0.0015 -0.0026
                                            0.0012  0.0030
   2. (1.86957) BD ( 2)Ga   1 - P   3
               ( 16.78%)   0.4096*Ga   1 s(  0.02%)p99.99( 99.60%)d14.32(  0.34%)
                                                  f 1.20(  0.03%)
                                            0.0000 -0.0000  0.0000 -0.0101 -0.0117
                                           -0.0006  0.0000 -0.0002 -0.2297  0.0062
                                            0.0030 -0.0000  0.0007  0.8142  0.0080
                                           -0.0127 -0.0000  0.0005  0.5292  0.0046
                                           -0.0070  0.0008  0.0006  0.0203  0.0040
                                            0.0010 -0.0127  0.0206  0.0050 -0.0008
                                            0.0243 -0.0009 -0.0028 -0.0014  0.0105
                                           -0.0274 -0.0048 -0.0014  0.0214 -0.0202
                                           -0.0060  0.0075 -0.0044 -0.0045  0.0110
                                            0.0016 -0.0081 -0.0008
               ( 83.22%)   0.9123* P   3 s(  0.01%)p 1.00( 99.42%)d 0.01(  0.55%)
                                                  f 0.00(  0.02%)
                                            0.0000  0.0000 -0.0079 -0.0017  0.0001
                                           -0.0001 -0.2364 -0.0002  0.0003  0.0002
                                            0.0002  0.8308 -0.0106 -0.0008 -0.0005
                                            0.0001  0.4980 -0.0008  0.0001 -0.0004
                                            0.0094  0.0003 -0.0145  0.0015  0.0349
                                           -0.0022  0.0300 -0.0024  0.0555 -0.0038
                                            0.0049 -0.0032 -0.0028  0.0092  0.0028
                                           -0.0075  0.0025

   3. (1.95417) BD ( 1) P   2 - P   3
               ( 46.33%)   0.6807* P   2 s( 11.98%)p 7.26( 87.02%)d 0.08(  0.99%)
                                                  f 0.00(  0.02%)
                                            0.0000 -0.0002 -0.3460 -0.0102  0.0023
                                            0.0002  0.8298 -0.0111 -0.0174  0.0001
                                           -0.0000  0.0716 -0.0125  0.0028  0.0003
                                            0.0001  0.4191  0.0122 -0.0133 -0.0009
                                           -0.0148  0.0040 -0.0626  0.0100  0.0010
                                           -0.0046 -0.0717  0.0078  0.0197 -0.0013
                                           -0.0057  0.0005 -0.0016  0.0089  0.0008
                                            0.0073  0.0003
               ( 53.67%)   0.7326* P   3 s( 13.36%)p 6.41( 85.67%)d 0.07(  0.91%)
                                                  f 0.00(  0.05%)
                                           -0.0000  0.0004 -0.3653 -0.0140 -0.0000
                                           -0.0000 -0.8417 -0.0254 -0.0017  0.0001
                                           -0.0001 -0.0131 -0.0075 -0.0010 -0.0001
                                            0.0000 -0.3840 -0.0007  0.0009  0.0003
                                            0.0095 -0.0039 -0.0754 -0.0018  0.0111
                                           -0.0043 -0.0548 -0.0093  0.0043  0.0073
                                            0.0066  0.0047  0.0037 -0.0119  0.0016
                                           -0.0149 -0.0078
```

Figure S21. Excerpt from NBO analysis table for **1a**

Atom	No	Natural Charge	Natural Population: Core	Valence	Rydberg	Total
Ga	1	1.31367	27.98768	1.67053	0.02811	29.68633
P	2	0.91665	9.99830	4.02343	0.06162	14.08335
P	3	-0.78927	9.99817	5.74602	0.04508	15.78927
N	4	-0.78278	1.99948	5.74415	0.03916	7.78278
N	5	-0.76460	1.99933	5.72980	0.03546	7.76460
N	6	-0.81248	1.99948	5.76881	0.04419	7.81248
N	7	-0.80021	1.99932	5.76424	0.03665	7.80021
C	8	0.14238	1.99852	3.83824	0.02086	5.85762
C	9	-0.06531	1.99876	4.04983	0.01672	6.06531

Figure S22. Excerpt from NPA analysis table for **1a**

3.2.2. **1b**

```
     (Occupancy)   Bond orbital/ Coefficients/ Hybrids
 ---------------------------------------------------------------------------------
   1. (1.97160) BD ( 1)Ga   1 - P   3
               ( 40.40%)   0.6356*Ga   1 s( 83.97%)p 0.19( 15.89%)d 0.00(  0.11%)
                                                f 0.00(  0.03%)
                                            -0.0000  0.0000 -0.0000 -0.9163 -0.0069
                                             0.0030  0.0000  0.0003  0.2911  0.0238
                                            -0.0009 -0.0000 -0.0003 -0.1815 -0.0332
                                            -0.0006 -0.0000 -0.0003 -0.1931 -0.0471
                                            -0.0004  0.0002 -0.0066 -0.0042 -0.0048
                                             0.0006 -0.0225 -0.0059 -0.0037  0.0003
                                             0.0034  0.0105  0.0067  0.0003 -0.0143
                                            -0.0030  0.0012 -0.0001  0.0090  0.0011
                                            -0.0005  0.0044  0.0043 -0.0065  0.0003
                                             0.0130 -0.0027 -0.0057
               ( 59.60%)   0.7720* P   3 s( 14.55%)p 5.81( 84.54%)d 0.06(  0.91%)
                                                f 0.00(  0.00%)
                                            -0.0000  0.0002 -0.3813  0.0067 -0.0000
                                            -0.0001 -0.4299 -0.0009  0.0003 -0.0007
                                             0.0001  0.5151 -0.0035  0.0000  0.0007
                                             0.0000  0.6287 -0.0027  0.0006  0.0007
                                             0.0329  0.0007  0.0474  0.0038 -0.0669
                                             0.0003  0.0121  0.0020 -0.0339  0.0016
                                            -0.0002  0.0005  0.0017 -0.0017 -0.0026
                                            -0.0004  0.0036
   2. (1.88494) BD ( 2)Ga   1 - P   3
               ( 17.26%)   0.4154*Ga   1 s(  0.11%)p99.99( 99.53%)d 3.14(  0.33%)
                                                f 0.28(  0.03%)
                                            -0.0000  0.0000 -0.0001 -0.0312  0.0093
                                            -0.0001  0.0000 -0.0001 -0.2483  0.0079
                                             0.0038  0.0000 -0.0005 -0.8058 -0.0128
                                             0.0135 -0.0000  0.0004  0.5328 -0.0018
                                            -0.0056  0.0006  0.0029  0.0197  0.0035
                                            -0.0008  0.0130 -0.0202 -0.0045 -0.0008
                                             0.0237  0.0012 -0.0016  0.0014 -0.0114
                                             0.0264  0.0036  0.0012 -0.0218  0.0193
                                             0.0067  0.0072 -0.0040  0.0030  0.0114
                                            -0.0018 -0.0092  0.0022
               ( 82.74%)   0.9096* P   3 s(  0.05%)p99.99( 99.39%)d10.57(  0.54%)
                                                f 0.40(  0.02%)
                                            -0.0000 -0.0000  0.0226  0.0019 -0.0002
                                            -0.0001 -0.2913 -0.0014 -0.0000  0.0003
                                            -0.0001 -0.8202  0.0072  0.0012  0.0005
                                             0.0000  0.4861 -0.0017  0.0001 -0.0002
                                             0.0098 -0.0003  0.0183 -0.0007  0.0360
                                            -0.0028 -0.0281  0.0023 -0.0536  0.0036
                                             0.0050 -0.0035  0.0016  0.0091 -0.0029
                                            -0.0084 -0.0020
```

Figure S23. Excerpt from NBO analysis of **1b**

Atom	No	Natural Charge	Natural Population Core	Valence	Rydberg	Total
Ga	1	1.30243	27.98774	1.68178	0.02806	29.69757
P	2	0.95715	9.99820	3.98143	0.06323	14.04285
P	3	-0.79942	9.99815	5.75710	0.04417	15.79942
N	4	-0.83951	1.99952	5.79920	0.04079	7.83951
N	5	-0.76838	1.99933	5.73365	0.03540	7.76838
N	6	-0.85474	1.99950	5.81190	0.04334	7.85474
N	7	-0.79390	1.99931	5.75758	0.03701	7.79390
C	8	0.34002	1.99875	3.64314	0.01808	5.65998
C	9	0.18026	1.99858	3.80047	0.02069	5.81974
C	10	0.17445	1.99856	3.80611	0.02087	5.82555
C	11	-0.20637	1.99906	4.19183	0.01548	6.20637

Figure S24. Excerpt from the NPA analysis of **1b**

3.3. Wiberg bond indices

Wiberg bond index (WBI) was calculated in the natural atomic orbital basis. Su and co-workers have investigated R_2E_{13}=$E_{15}R'_2$ multiple bonds computationally.[8a] In the case of E_{13} = Ga, E_{15} = P they found no significant π-contribution to the bond, and a resultant WBI of 0.99. The WBI of the Ga=P bond of **1b**$_{\mathbf{DFT}}$ is 1.49, consistent with significant π-contribution. The NBO analysis on Su and co-workers' system implies a σ-bond with polarity (27.82% Ga; 72.18% P) and occupancy (1.91e) comparable to that obtained for **1b**$_{\mathbf{DFT}}$ (40.4% Ga; 59.6% P and 1.97e), the discrepancy in polarity may be attributed to the π-contribution to the bonding in Su's system, which is highly polarised in the case of **1b**$_{\mathbf{DFT}}$. The WBI for the Schulz group's gallaarsene Ga=As bond is 1.65.[8b]

Wiberg bond index matrix in the NAO basis:

Atom	1	2	3	4	5	6	7	8	9
1. Ga	0.0000	0.0432	1.4890	0.0179	0.3340	0.0155	0.3352	0.0103	0.0004
2. P	0.0432	0.0000	1.0128	0.7603	0.0061	0.7488	0.0022	0.0009	0.0101
3. P	1.4890	1.0128	0.0000	0.0524	0.0310	0.0554	0.0587	0.0051	0.0030
4. N	0.0179	0.7603	0.0524	0.0000	0.0011	0.0253	0.0009	0.0003	0.9996
5. N	0.3340	0.0061	0.0310	0.0011	0.0000	0.0017	0.0490	1.3963	0.0000
6. N	0.0155	0.7488	0.0554	0.0253	0.0017	0.0000	0.0006	0.0002	0.0059
7. N	0.3352	0.0022	0.0587	0.0009	0.0490	0.0006	0.0000	0.0523	0.0000
8. C	0.0103	0.0009	0.0051	0.0003	1.3963	0.0002	0.0523	0.0000	0.0000
9. C	0.0004	0.0101	0.0030	0.9996	0.0000	0.0059	0.0000	0.0000	0.0000
10. C	0.0003	0.0089	0.0021	0.0054	0.0000	0.9855	0.0000	0.0000	0.0004
11. C	0.0011	0.0249	0.0033	0.9524	0.0001	0.0133	0.0001	0.0000	0.0092
12. H	0.0007	0.0130	0.0012	0.0094	0.0000	0.0094	0.0000	0.0000	0.0006

Figure S25. Excerpt displaying the WBI of **1b**$_{\mathbf{DFT}}$. Ga=P bond is between atom 1 and 3.

3.4. Kohn-Sham orbitals of **1b$_{DFT}$**

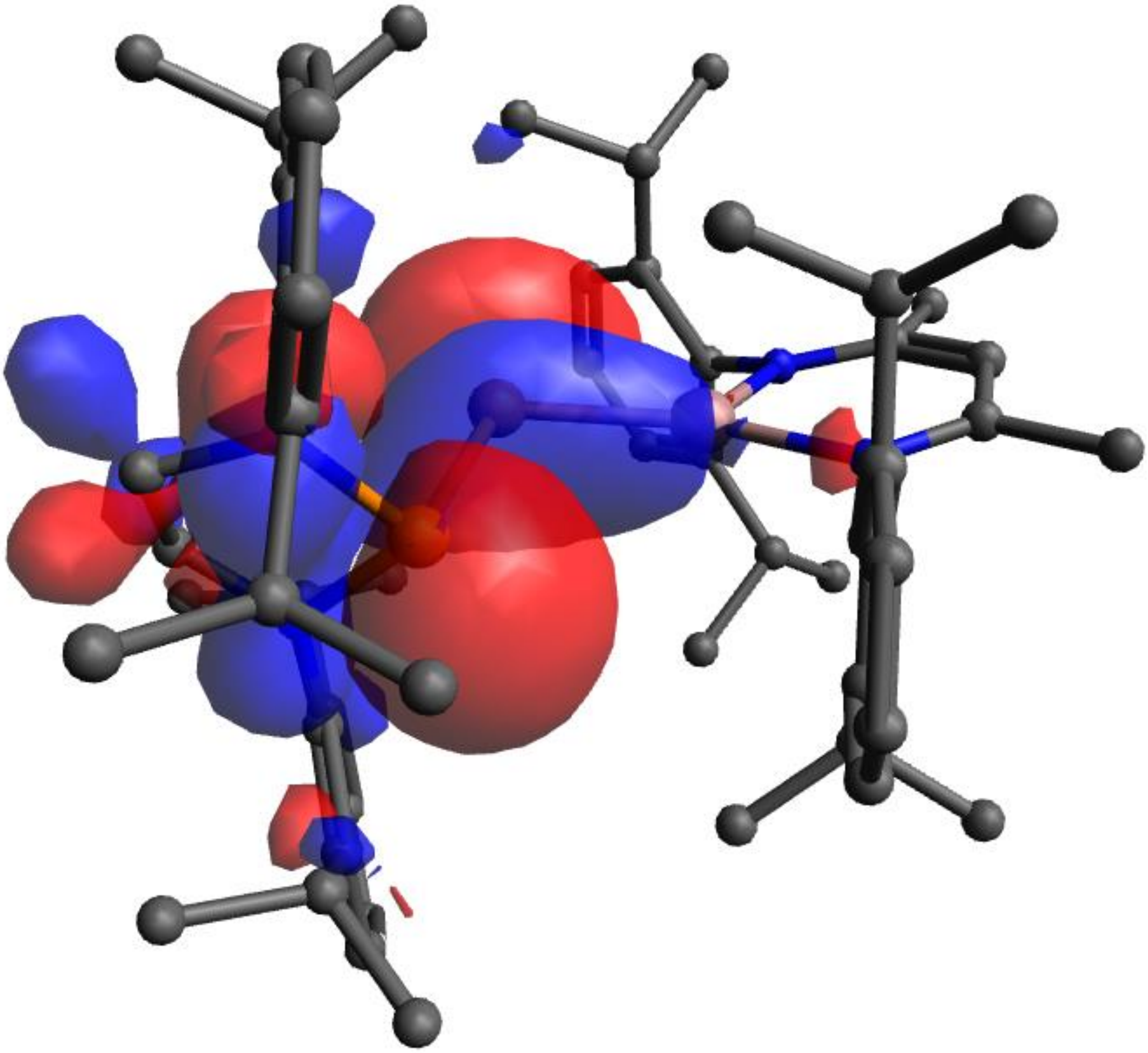

Figure S26. HOMO of **1b$_{DFT}$**

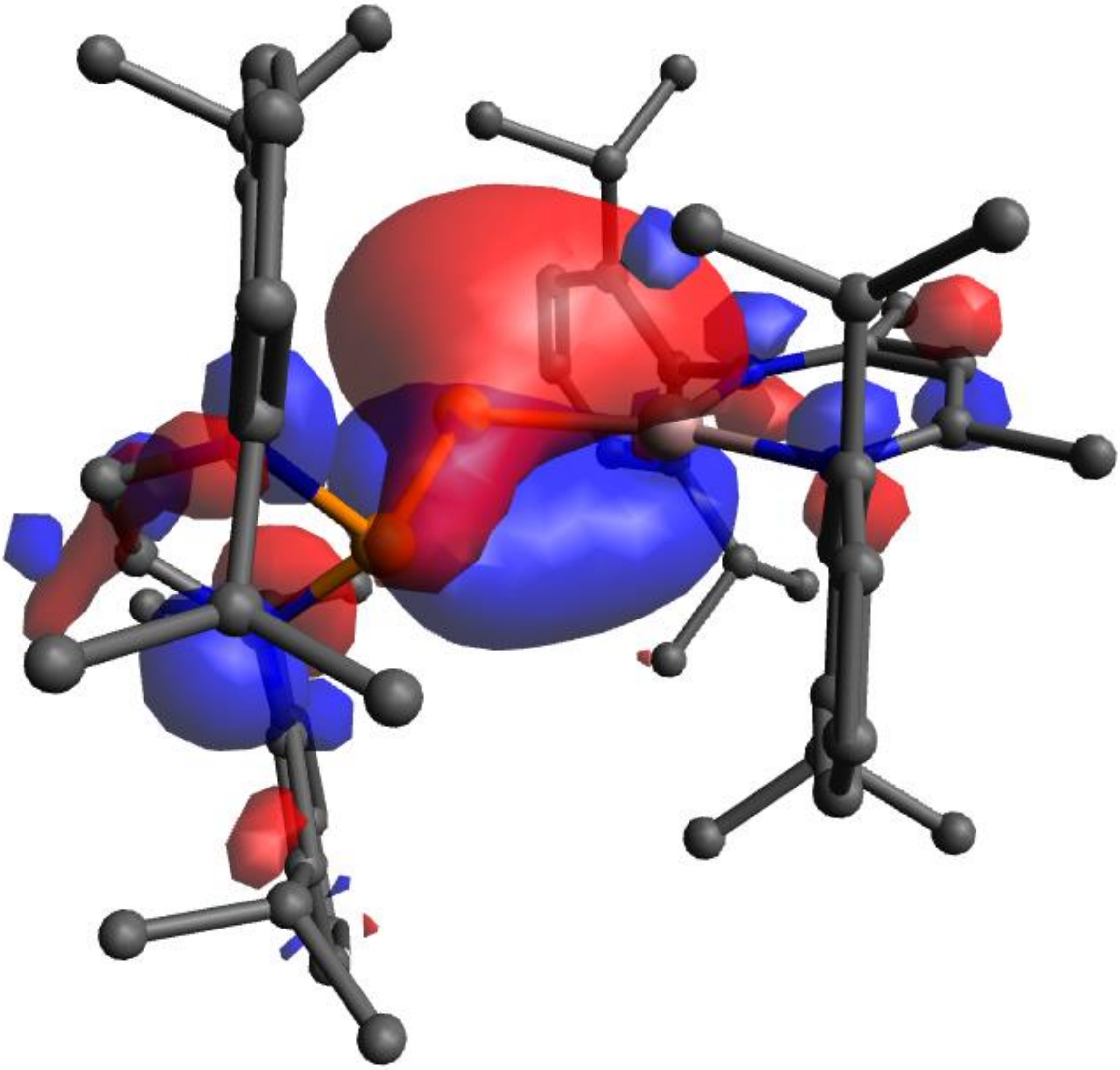

Figure S27. HOMO−1 of $\mathbf{1b}_{\mathbf{DFT}}$

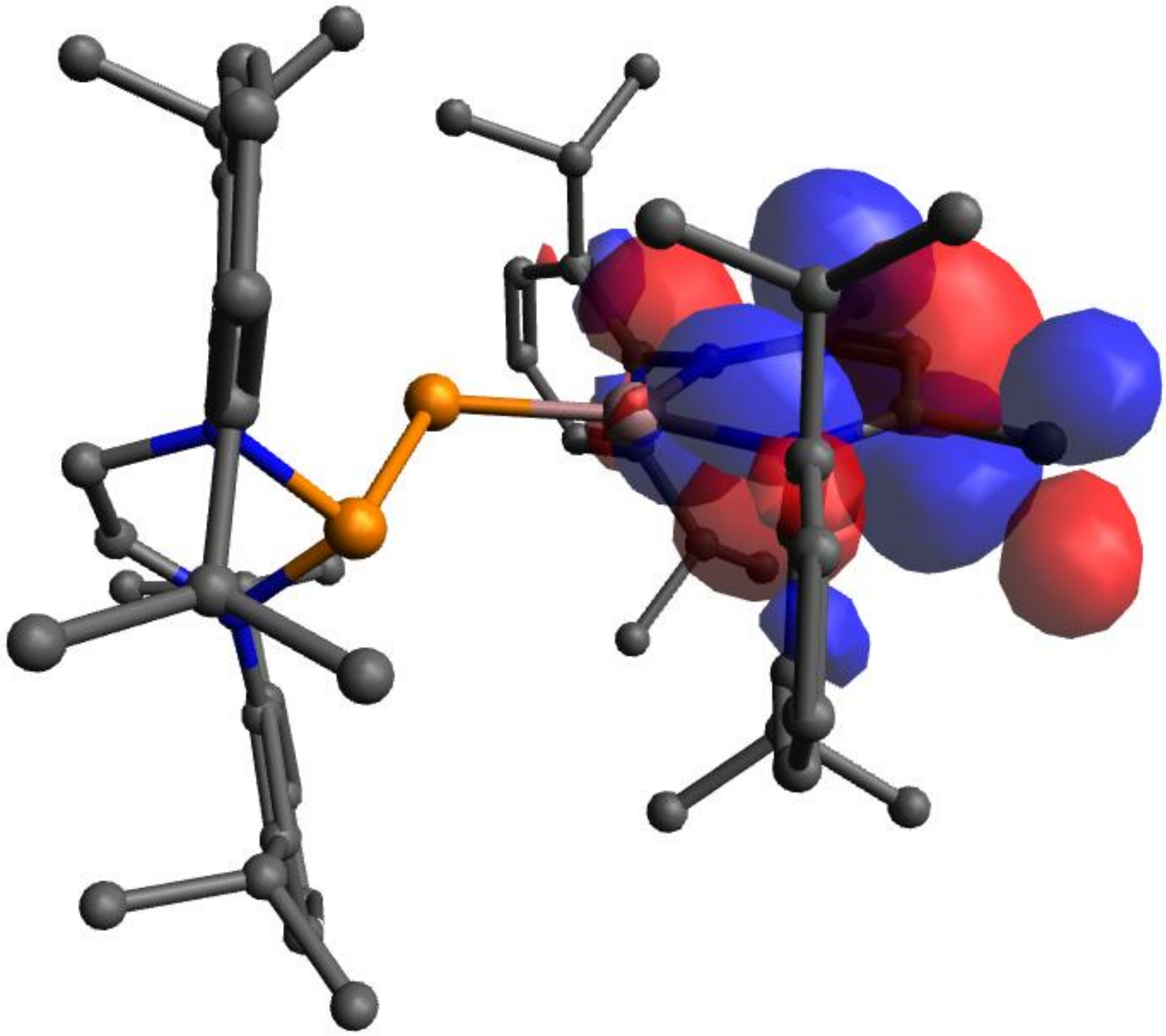

Figure S28. LUMO of **1b**$_{\mathbf{DFT}}$

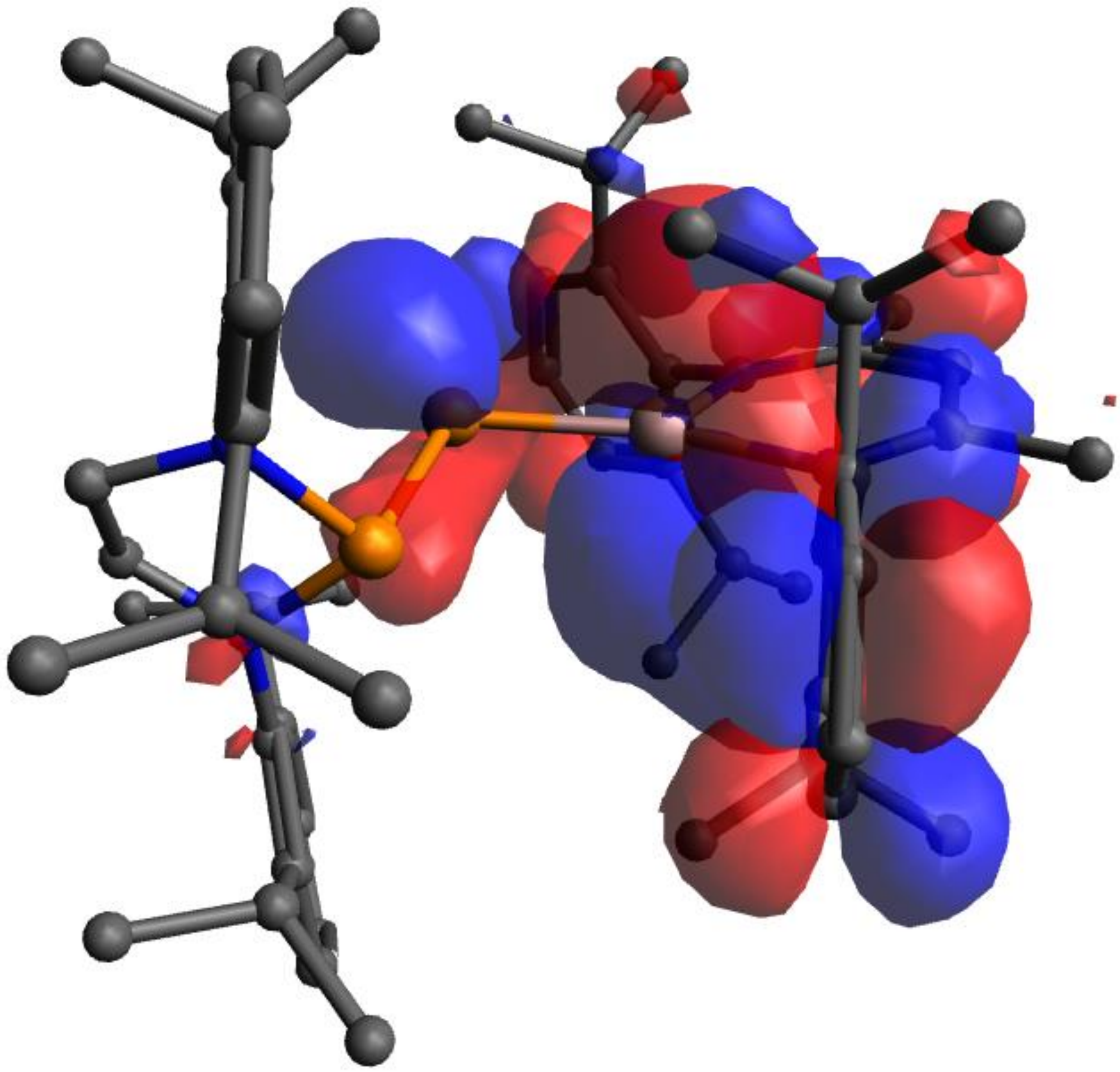

Figure S29. LUMO+1 of **1b**$_{\mathbf{DFT}}$

3.5.Optimised structures and coordinates

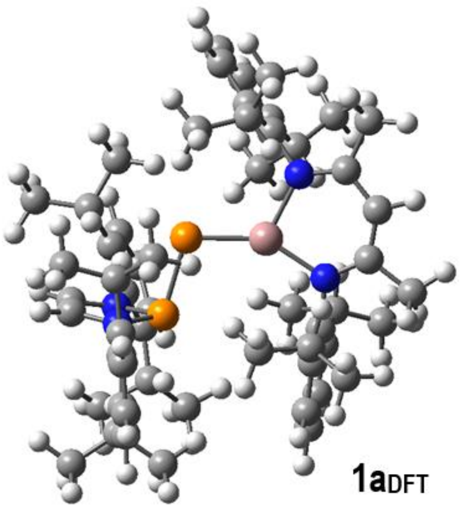

$1a_{DFT}$

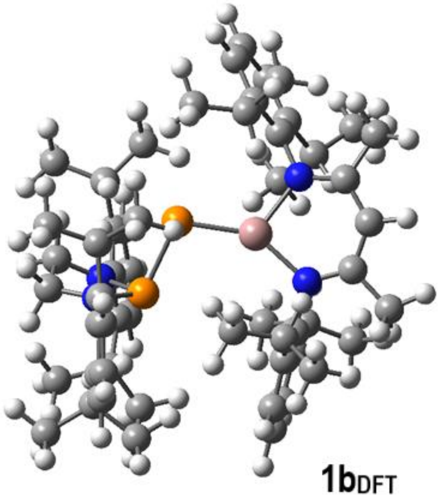

$1b_{DFT}$

Coordinates of **1a**$_{DFT}$

Ga	-1.49865500	-0.81743000	0.22204800
P	1.74160500	0.53199400	-0.31606500
P	-0.32009000	0.39239600	-1.19614900
N	2.10422300	2.25081800	-0.60692800
N	-3.45460000	-0.99389800	-0.16185700
N	2.80582400	0.13915700	-1.66541100
N	-1.49388200	-1.96808900	1.81043500
C	-0.30305900	-1.95104300	2.63368200
C	3.17037500	1.28688700	-2.40192400
H	3.72620300	1.18009400	-3.33023600
C	-4.05244100	-0.04658100	-1.07650700
C	-3.77192500	-2.72954500	1.49172300
H	-4.51395400	-3.42100900	1.88528500
C	2.76162400	2.43271200	-1.83340600
H	2.93319500	3.43761500	-2.20984500
C	0.60693000	-3.99456000	1.33502400
H	-0.42267700	-3.98417700	0.94858000

C	-4.50151100	1.19029000	-0.55318400
C	3.29652600	-1.95976800	-2.80353100
C	-4.22526100	-1.88540900	0.45564100
C	0.72103900	-2.89943900	2.39479300
C	0.55192800	4.15292600	-0.59892700
C	1.48920800	3.33733300	0.08224900
C	3.61824500	-1.04151800	-1.77033000
C	-4.18023700	-0.35319500	-2.45342200
C	-2.54163300	-2.72692300	2.16244100
C	4.74956800	-1.26536900	-0.94260800
C	-0.18096800	-0.97424900	3.65340900
C	-5.07985500	2.11163000	-1.43612800
H	-5.43323600	3.07109900	-1.05190000
C	3.02228900	2.93527600	2.12148000
H	2.99325600	1.87469300	1.83738900
C	-5.68828200	-1.99521800	0.09522200
H	-6.21342400	-1.05285700	0.31208600
H	-6.17208500	-2.80380700	0.65703400
H	-5.81916500	-2.17974400	-0.97794900
C	0.16034900	3.92889800	-2.06105600
H	0.58908500	2.96928600	-2.38128800
C	5.24628700	-0.26313900	0.09136600
H	4.56767200	0.59792100	0.07618100
C	-1.27965100	0.03569500	3.97861900
H	-2.11695400	-0.13813900	3.28753800
C	1.86352600	3.64434700	1.42599000
C	0.86235300	-5.39893100	1.91263700
H	1.90558500	-5.51923000	2.24519500
H	0.67554100	-6.16618400	1.14410100

H	0.21429800	-5.62087000	2.77537800
C	-3.70154900	-1.66567600	-3.07493100
H	-3.29412500	-2.29445500	-2.26955000
C	5.49156200	-2.44207000	-1.12695800
H	6.35890200	-2.63077100	-0.48903100
C	-4.39886300	1.55224000	0.92846900
H	-3.94174800	0.70065600	1.45393100
C	5.16876400	-3.35863800	-2.12333100
H	5.77002500	-4.26189000	-2.25650800
C	1.53967500	-3.72617800	0.14377500
H	1.32107000	-2.75823400	-0.32872200
H	1.42742300	-4.51046800	-0.62180100
H	2.59644900	-3.71154500	0.45286700
C	4.08893900	-3.10427700	-2.96402100
H	3.85506700	-3.81148700	-3.76349700
C	2.15216100	-1.70157000	-3.77879800
H	1.55160500	-0.88023800	-3.36249500
C	1.00071300	-0.95204700	4.40683400
H	1.11946300	-0.20511900	5.19506800
C	1.88109400	-2.82680100	3.17830300
H	2.68782100	-3.54252800	3.00658500
C	-2.42611000	-3.62851900	3.36802700
H	-1.62565600	-4.36824200	3.23242800
H	-3.36827300	-4.15976100	3.55147900
H	-2.16523200	-3.05153800	4.26642500
C	1.23308100	4.71402900	2.07190700
H	1.50021800	4.95298500	3.10266900
C	-2.56649400	-1.41962900	-4.08427500
H	-2.90849300	-0.79097900	-4.92268900

H	-2.21681000	-2.37575900	-4.50658500
H	-1.71176000	-0.91726400	-3.60779100
C	6.64378700	0.26511700	-0.28066600
H	7.40236900	-0.53487800	-0.26399800
H	6.96504000	1.04219100	0.43090500
H	6.64544400	0.70880800	-1.28837600
C	-1.35844700	3.81790300	-2.26354500
H	-1.76519600	2.96212100	-1.70797400
H	-1.58741000	3.66357200	-3.33066800
H	-1.88883900	4.72844400	-1.94115400
C	-5.21675400	1.82765700	-2.79254100
H	-5.67266900	2.55933000	-3.46390000
C	-4.77073700	0.60646900	-3.28834700
H	-4.87729800	0.39187400	-4.35416400
C	0.74760600	5.02643400	-2.96955000
H	0.33108300	6.01786800	-2.72550100
H	0.51539800	4.81920900	-4.02724200
H	1.84228600	5.10031000	-2.87322500
C	2.02655400	-1.86314000	4.17189600
H	2.94138300	-1.82478200	4.76797500
C	-0.01212900	5.24157300	0.08523400
H	-0.72558700	5.88400100	-0.43590900
C	0.29987000	5.51268000	1.41152800
H	-0.16791100	6.35520800	1.92744500
C	-4.84331900	-2.45458600	-3.74381800
H	-5.67323100	-2.66443400	-3.05148500
H	-4.47020900	-3.42077700	-4.12048400
H	-5.26375600	-1.90838900	-4.60346900
C	5.22680300	-0.84044900	1.51593200

H	4.21071600	-1.14727600	1.80560300
H	5.57402600	-0.08935400	2.24391400
H	5.88882500	-1.71784400	1.60774200
C	4.35813100	3.52175800	1.62353100
H	4.46213200	3.42639400	0.53357400
H	5.21179500	3.00705700	2.09324700
H	4.42811300	4.59234500	1.87866800
C	-1.82826800	-0.15364500	5.40507600
H	-2.21483900	-1.17196100	5.56788400
H	-2.65207500	0.55274800	5.59778900
H	-1.05083000	0.03050200	6.16394000
C	-0.80318800	1.48261400	3.76792100
H	0.00714000	1.74149800	4.46799200
H	-1.62850900	2.19093900	3.94391600
H	-0.42843500	1.64554000	2.74604100
C	1.22428600	-2.91474800	-3.93829500
H	1.74831900	-3.78832900	-4.36046200
H	0.39590000	-2.67100700	-4.62232700
H	0.78760700	-3.21424500	-2.97340900
C	-5.78430000	1.77320600	1.56141900
H	-6.30362900	2.63391500	1.11016700
H	-5.68724700	1.97548500	2.64057700
H	-6.43618200	0.89300800	1.44182400
C	2.68615300	-1.24393000	-5.14918500
H	3.31341000	-0.34345900	-5.06146800
H	1.85332700	-1.00713000	-5.83194300
H	3.29823800	-2.02864300	-5.62539100
C	2.96449900	2.98463200	3.65309300
H	3.12824800	4.00171100	4.04611100

H	3.75558200	2.34592400	4.07739500
H	1.99786400	2.62430000	4.03803600
C	-3.48936000	2.77049900	1.16065600
H	-2.47767600	2.59570600	0.76736600
H	-3.40490200	2.98848300	2.23760100
H	-3.88997900	3.67246100	0.67099400

Coordinates of $\mathbf{1b}_{\mathbf{DFT}}$

Ga	1.48746800	-0.85780500	-0.12898400
P	-1.69309300	0.69190500	0.34819200
P	0.32273300	0.34377700	1.30059700
N	-1.94864500	2.39248200	0.62328800
N	3.45804400	-1.02677800	0.14161900
N	-2.82514200	0.35463100	1.63534700
N	1.40536500	-2.10899700	-1.65060200
C	4.19807300	-1.96385600	-0.44910600
C	-1.45959900	3.42754800	-0.23232100
C	-3.50495500	-0.89238800	1.81027300
C	-2.45764500	2.67979700	1.96460300
H	-2.97545900	3.65107500	1.97253800
H	-1.66194200	2.71388100	2.73062500
C	3.69307000	-2.87158800	-1.40030300
H	4.41025700	-3.59593400	-1.78169500
C	-3.41063300	1.53303200	2.27157800
H	-3.51715600	1.38682900	3.36003800
H	-4.41985800	1.74429700	1.86567700
C	-2.10949600	3.63889800	-1.48458500
C	-3.15470800	-1.72803000	2.90626400
C	2.43005100	-2.89321300	-2.01119700

C	4.10956400	-0.00973000	0.93709700
C	0.17331200	-2.17521800	-2.40510200
C	-4.57157600	-1.26599700	0.94821600
C	-2.09072500	-1.32736000	3.92361500
H	-1.57122100	-0.44849800	3.51656400
C	-0.38268700	4.27251200	0.15535200
C	-0.12382300	5.24466200	2.48044800
H	0.15242300	6.24622600	2.11014800
H	0.35169100	5.11029900	3.46633700
H	-1.21441300	5.23962200	2.62726300
C	0.33600900	4.14831500	1.49904300
H	0.08134500	3.16898900	1.92162700
C	4.14280300	-0.12728400	2.34784700
C	-3.36041600	2.85220800	-1.87211400
H	-3.25808700	1.84334100	-1.44969400
C	3.55340900	-1.32328700	3.09609100
H	2.92121400	-1.88249500	2.39018900
C	-3.84549600	-2.93574300	3.08589900
H	-3.58310800	-3.58303800	3.92645600
C	-0.78215300	-3.17573300	-2.09706700
C	5.34705800	1.99180200	2.44659500
H	5.83017400	2.77388400	3.03777300
C	5.68092400	-2.05002200	-0.16329400
H	6.22339600	-1.29096500	-0.74893200
H	6.07231200	-3.03577300	-0.44706200
H	5.91015900	-1.85738600	0.89165400
C	4.68798300	1.10374200	0.27558600
C	-5.08159700	-0.35654800	-0.16588000
H	-4.45132500	0.54209300	-0.17189200

C	0.37139000	1.25778500	-3.70430700
H	-0.42518700	1.41729700	-4.44858100
H	1.14662100	2.01880700	-3.88638800
H	-0.05861500	1.44411300	-2.70898700
C	-1.64585100	4.65647000	-2.32831600
H	-2.13450900	4.82243000	-3.28997000
C	-0.59121000	5.48698900	-1.95204900
H	-0.25253600	6.28431500	-2.61923700
C	0.96819200	-0.15547800	-3.81066600
H	1.79837200	-0.21254300	-3.09091200
C	4.65250600	-2.28351100	3.59260400
H	5.27004800	-2.67429200	2.76972300
H	4.20391600	-3.14693200	4.11046300
H	5.32710900	-1.78028900	4.30490700
C	-2.73148100	-0.92652200	5.26688300
H	-3.47774500	-0.12628200	5.14190500
H	-1.96392400	-0.56600000	5.97184300
H	-3.24329100	-1.78198900	5.73930600
C	2.25035300	-3.86046000	-3.16010500
H	1.46071200	-4.59369000	-2.94420500
H	3.18338200	-4.39930800	-3.36602200
H	1.93878600	-3.33105400	-4.07213500
C	-0.04778100	-1.23602600	-3.44129000
C	0.01778600	5.29879300	-0.71632800
H	0.83636700	5.95872600	-0.41996000
C	-0.57937600	-4.19275800	-0.97377000
H	0.47292600	-4.14239400	-0.65751100
C	-5.22117100	-2.49122200	1.16240900
H	-6.03796800	-2.78786500	0.49897400

C	-4.86221000	-3.32647400	2.21755500
H	-5.38454800	-4.27468200	2.37204000
C	4.77651400	0.88957300	3.07796000
H	4.82022000	0.81674200	4.16667000
C	5.29715300	2.09451400	1.05818900
H	5.74178100	2.96493700	0.57035900
C	-3.55722900	2.67630200	-3.38440900
H	-3.79703400	3.62506700	-3.89204400
H	-4.39840500	1.99048700	-3.57501000
H	-2.66199600	2.25155800	-3.86345200
C	-1.96016900	-3.21470600	-2.85581700
H	-2.71391600	-3.97330600	-2.63460200
C	1.86683600	4.15739300	1.37129600
H	2.21120200	3.36070400	0.69850200
H	2.32765300	3.97890700	2.35609900
H	2.25311700	5.12048400	0.99872600
C	-1.03126700	-2.41963400	4.13676100
H	-1.46309800	-3.33868200	4.56687500
H	-0.25562800	-2.06475500	4.83364100
H	-0.53624300	-2.68128600	3.18998100
C	-6.52874100	0.09818600	0.10327000
H	-7.23119800	-0.75140500	0.08743100
H	-6.85929900	0.81531600	-0.66614700
H	-6.62300800	0.58756200	1.08544300
C	-4.95636500	-1.00250100	-1.55459100
H	-3.91420700	-1.27679400	-1.77131400
H	-5.29265500	-0.30322900	-2.33813600
H	-5.57461100	-1.91210600	-1.63820800
C	-1.43568100	-3.84174100	0.25501900

H	-1.21263200	-2.83163700	0.62782500
H	-1.24821300	-4.55471700	1.07419700
H	-2.51112200	-3.87648500	0.02004800
C	-0.84551900	-5.64050900	-1.42460400
H	-1.90824000	-5.80760300	-1.66159600
H	-0.57975900	-6.34346300	-0.61878000
H	-0.26044500	-5.91382700	-2.31760200
C	3.66114200	2.36074100	-1.67755900
H	2.64367000	2.14512900	-1.31810800
H	3.62669700	2.43952500	-2.77666200
H	3.94952700	3.34717400	-1.28065600
C	4.66071300	1.27238800	-1.24531300
H	4.31454800	0.32699400	-1.68726600
C	1.57043100	-0.39133300	-5.20900600
H	0.79868900	-0.33162200	-5.99365600
H	2.05031100	-1.37929700	-5.29034100
H	2.33299300	0.37180400	-5.43505300
C	2.65099500	-0.89546200	4.26480400
H	3.22035500	-0.38521100	5.05860700
H	2.18045400	-1.78114800	4.72008500
H	1.85134400	-0.22423200	3.91923000
C	-4.60821200	3.50040600	-1.24172200
H	-4.51817900	3.56815600	-0.14744800
H	-5.51476900	2.91630400	-1.47123500
H	-4.75657000	4.52194100	-1.62996700
C	-2.19189700	-2.30373700	-3.88492300
H	-3.11755200	-2.35500400	-4.46340600
C	-1.24388300	-1.32443800	-4.16887000
H	-1.43444700	-0.61191500	-4.97482200

C	6.05540500	1.55595000	-1.83286000
H	6.44117500	2.53858500	-1.51782900
H	6.01268700	1.56154600	-2.93409200
H	6.79431400	0.79836700	-1.52676400

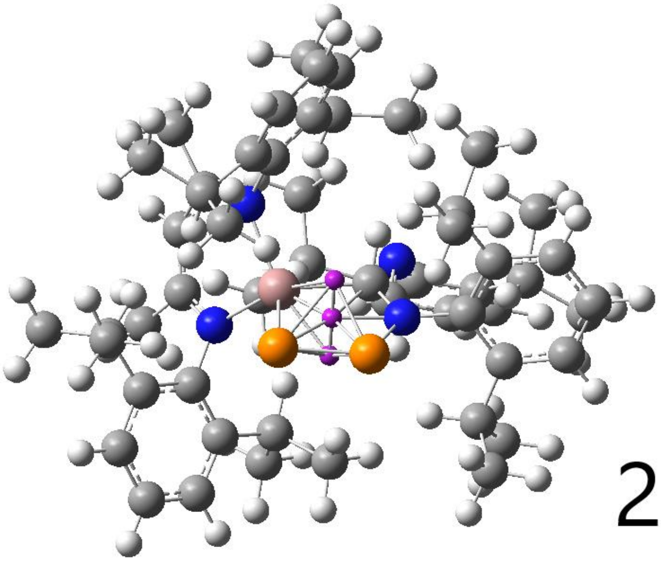

2'

Coordinates of **2**$_{\mathbf{DFT}}$

C	-0.79030000	0.48132800	-0.37484800
H	-0.86420100	1.34903500	0.29041600
C	-1.65470600	-0.60317500	0.16099600
H	-1.89730800	-1.41392200	-0.53990300
C	-2.46529000	1.37475300	-2.03809300
C	-2.77951100	2.71960300	-1.71557100
C	-4.08225000	3.17462800	-1.95030000
H	-4.34101800	4.19859400	-1.70203000
C	-5.04626300	2.35095800	-2.52308300
H	-6.05055700	2.72609200	-2.69879500
C	-4.70480700	1.05968700	-2.90453500
H	-5.44426600	0.43589000	-3.39748100
C	-3.41858300	0.54843700	-2.68295600
C	-1.72476700	3.71894400	-1.24139500

H	-0.87217100	3.16619600	-0.83757000
C	-1.19848200	4.53707100	-2.44136000
H	-0.79146900	3.88916400	-3.22292300
H	-0.40599000	5.22060000	-2.11739500
H	-2.00287500	5.13340500	-2.88628600
C	-2.23526200	4.67083800	-0.14600400
H	-2.97750200	5.37522500	-0.53643400
H	-1.40397500	5.26045400	0.24974600
H	-2.69254600	4.12829700	0.68516900
C	-3.07907600	-0.83225800	-3.23609300
H	-2.08296900	-1.09105800	-2.87732800
C	-3.01251500	-0.79078200	-4.77710200
H	-3.98921400	-0.54592400	-5.20916000
H	-2.70583100	-1.76555800	-5.17240800
H	-2.29440200	-0.04161200	-5.12186500
C	-4.04348800	-1.93642500	-2.76865300
H	-4.05740200	-2.03056100	-1.67911400
H	-3.73858200	-2.90183100	-3.18674500
H	-5.07031800	-1.75021200	-3.10035300
C	-2.92352500	-1.67359900	1.81300100
C	-2.37032400	-2.67966300	2.64161400
C	-3.20719500	-3.69906600	3.10824300
H	-2.79410900	-4.48755500	3.72850200
C	-4.56277400	-3.72114800	2.79686200
H	-5.19572500	-4.52445300	3.16284400
C	-5.10600700	-2.69494900	2.03066900
H	-6.16891900	-2.70390300	1.81550000
C	-4.31554700	-1.65142300	1.53382500
C	-0.91478900	-2.59988000	3.09351500

H	-0.33675300	-2.11436400	2.30101700
C	-0.80626100	-1.69894300	4.34154200
H	-1.22863500	-0.71045600	4.14374900
H	0.24075700	-1.57278500	4.64011800
H	-1.35202500	-2.13909700	5.18373700
C	-0.27047300	-3.96725800	3.36680000
H	-0.71804500	-4.46519600	4.23351800
H	0.79371500	-3.84196700	3.58882900
H	-0.36480800	-4.64040800	2.50869400
C	-4.95847100	-0.47427000	0.80031500
H	-4.28395000	-0.15665500	0.00049600
C	-5.11661900	0.72537700	1.75770000
H	-5.80377100	0.47774200	2.57494700
H	-5.51847100	1.59323900	1.22310300
H	-4.15652200	1.00751700	2.19449200
C	-6.30793500	-0.80062000	0.14259400
H	-6.25054100	-1.68704900	-0.49647700
H	-6.62421800	0.04253400	-0.47904200
H	-7.09542200	-0.97026700	0.88536500
C	2.52832800	0.44099000	2.33857400
C	2.66445400	-0.96183300	2.42953000
H	3.04900300	-1.32589200	3.37385600
C	2.58568700	-1.90144200	1.40170300
C	3.20105900	1.20085300	3.46583800
H	2.93917200	2.25493700	3.49972000
H	4.28608600	1.12322800	3.33116300
H	2.96522200	0.73906600	4.42767400
C	3.05319300	-3.30760600	1.71628900
H	2.28577300	-4.03389000	1.43621000

H	3.26758300	-3.41514100	2.78056400
H	3.95245900	-3.56781900	1.15399700
C	1.72673400	2.51307700	1.42840300
C	0.69440500	3.02502200	2.26129600
C	0.52790300	4.41432600	2.32394400
H	-0.24562100	4.82167900	2.96719200
C	1.32784200	5.28277200	1.58976000
H	1.17647900	6.35619900	1.65760900
C	2.33285800	4.76556200	0.78259500
H	2.97196800	5.44394800	0.22642000
C	2.55992700	3.38642300	0.69096900
C	-0.20122200	2.15538500	3.14909200
H	-0.02156700	1.10575700	2.90368600
C	-1.69973100	2.42114700	2.91828200
H	-1.97187500	3.45052800	3.17457800
H	-2.29335600	1.75380100	3.54948000
H	-1.99173900	2.23227700	1.88493400
C	0.10757800	2.36484300	4.64832900
H	1.15541400	2.18580700	4.89560500
H	-0.50147300	1.68612800	5.25442800
H	-0.13302000	3.38850900	4.95561300
C	3.75627000	2.90341100	-0.12394700
H	3.70815100	1.81374900	-0.19482300
C	3.73648900	3.45800300	-1.55815300
H	2.79248600	3.21845100	-2.05175900
H	4.54435800	3.01205400	-2.14686500
H	3.87681700	4.54410600	-1.57815500
C	5.08053900	3.25600500	0.58380200
H	5.20204800	4.34057800	0.67773100

H	5.93338900	2.87322400	0.01280600
H	5.12778800	2.82958500	1.59082500
C	2.70961300	-2.43412700	-0.91603700
C	1.90945500	-3.42171700	-1.52652500
C	2.46373000	-4.20071400	-2.55016100
H	1.85374900	-4.96335600	-3.02516300
C	3.77641000	-4.01912500	-2.96585500
H	4.19021300	-4.63443800	-3.75942100
C	4.55736400	-3.04208400	-2.35740200
H	5.58252600	-2.90024600	-2.68631200
C	4.05160600	-2.23224600	-1.33485700
C	0.46205300	-3.66555500	-1.12036700
H	0.23131600	-3.00136100	-0.28166800
C	-0.47994500	-3.30090200	-2.27967900
H	-0.29113300	-2.27893500	-2.61839300
H	-1.52659900	-3.38680900	-1.97103900
H	-0.33121400	-3.96528600	-3.13774500
C	0.21073500	-5.10913700	-0.64614500
H	0.36787300	-5.83156000	-1.45420100
H	-0.82226600	-5.21981000	-0.29973700
H	0.87482800	-5.38836500	0.17798800
C	4.97959500	-1.18268900	-0.71626800
H	4.41079100	-0.60569400	0.01767600
C	5.50738100	-0.19326900	-1.77318100
H	6.15662500	-0.69059200	-2.50153300
H	6.10027200	0.59136100	-1.28990700
H	4.68978700	0.27982000	-2.32138000
C	6.16392100	-1.82784700	0.03317500
H	5.83305500	-2.48699900	0.84074900

H	6.79981200	-1.05318800	0.47556200
H	6.78768300	-2.42011100	-0.64474900
N	-1.12987200	0.86522500	-1.75812800
N	-2.09089700	-0.61055400	1.36522300
N	1.92504400	1.08013800	1.33633300
N	2.18587800	-1.60622400	0.15120000
P	1.86832000	0.54682200	-2.51984300
P	-0.03389600	1.02871500	-3.07179600
Ga	1.24566800	0.09639500	-0.30077700
Bq	0.40063300	0.56970700	-1.56702100
Bq	0.55579800	1.52622800	-1.32005600
Bq	0.24546700	-0.38681400	-1.81398600

4. References

[1] L. Liu, D. A. Ruiz, D. Munz, G. Bertrand, *Chem* **2016**, *1*, 147–153.

[2] Z. Li, X. Chen, M. Bergeler, M. Reiher, C. Y. Su, H. Grützmacher, *Dalt. Trans.* **2015**, *44*, 6431–6438.

[3] N. J. Hardman, B. E. Eichler, P. P. Power, *Chem. Commun.* **2000**, 1991–1992.

[4] Kost, D.; Carlson, E. H.; Raban, M. J. J. *Chem. Soc., Chem. Commun.* **1971**, 656.

[5] *CrysAlisPro*, Agilent Technologies, Version 1.171.35.8.

[6] (a) G. M. Sheldrick in SHELXL97, *Programs for Crystal Structure Analysis (Release 97-2)*, Institut für Anorganische Chemie der Universität, Tammanstrasse 4, D-3400 Göttingen, Germany, 1998; (b) G. M. Sheldrick, *Acta Crystallogr. Sect. A* 1990, **46**, 467−473; (c) G. M. Sheldrick, *Acta Crystallogr. Sect. A* 2008, **64**, 112−122.

[7] M. A. Petrie, P. P. Power, *Inorg. Chem.* **1993**, *32*, 1309–1312.

[8] a) T. W. Shih, M. C. Li, M. Der Su, *Inorg. Chem.* **2015**, *54*, 5154–5161. b) C. Helling, C. Wölper, S. Schulz, *J. Am. Chem. Soc.* **2018**, *140*, 5053–5056.
